# LncRNA SOX2‐OT regulates AKT/ERK and SOX2/GLI‐1 expression, hinders therapy, and worsens clinical prognosis in malignant lung diseases

**DOI:** 10.1002/1878-0261.12875

**Published:** 2020-12-25

**Authors:** Abril Marcela Herrera‐Solorio, Irlanda Peralta‐Arrieta, Leonel Armas López, Nallely Hernández‐Cigala, Criselda Mendoza Milla, Blanca Ortiz Quintero, Rodrigo Catalán Cárdenas, Priscila Pineda Villegas, Evelyn Rodríguez Villanueva, Cynthia G. Trejo Iriarte, Joaquín Zúñiga, Oscar Arrieta, Federico Ávila‐Moreno

**Affiliations:** ^1^ Biomedicine Research Unit (UBIMED) Lung Diseases and Cancer Epigenomics Laboratory Facultad de Estudios Superiores (FES) Iztacala National Autonomous University of Mexico (UNAM) Tlalnepantla de Baz Mexico; ^2^ National Institute of Respiratory Diseases (INER), Ismael Cosío Villegas Mexico City Mexico; ^3^ Thoracic Oncology Unit Laboratory of Personalized Medicine Instituto Nacional de Cancerología (INCAN) Mexico City Mexico; ^4^ Grupo de Investigación en Células Troncales e Ingeniería de Tejidos (GICTIT) Laboratorio de Investigación en Odontología Almaraz FES‐Iztacala National Autonomous University of México (UNAM) Tlalnepantla de Baz Mexico

**Keywords:** drug resistance, GLI‐1, LncRNA, lung adenocarcinoma, SOX2, SOX2‐OT

## Abstract

The involvement of LncRNA SOX2‐overlapping transcript (SOX2‐OT), SOX2, and GLI‐1 transcription factors in cancer has been well documented. Nonetheless, it is still unknown whether co‐expressed SOX2‐OT/SOX2 or SOX2‐OT/SOX2/GLI‐1 axes are epigenetically/transcriptionally involved in terms of resistance to oncology therapy and in poorer clinical outcomes for patients with lung cancer. We evaluated the role of SOX2‐OT/SOX2 and SOX2‐OT/SOX2/GLI‐1 axes using RT‐qPCR, western blot, immunofluorescence analyses, gene silencing, cellular cytotoxic, and ChIP‐qPCR assays on human cell lines, solid lung malignant tumors, and normal lung tissue. We detected that the SOX2‐OT/SOX2/GLI‐1 axis promotes resistance to tyrosine kinase inhibitor (TKI)‐erlotinib and cisplatin‐based therapy. Evidence from this study show that SOX2‐OT modulates the expression/activation of EGFR‐pathway members AKT/ERK. Further, both SOX2‐OT and GLI‐1 genes are epigenetically regulated at their promoter sequences, in an LncRNA SOX2‐OT‐dependent manner, mainly through modifying the enrichment of the activation histone mark H3K4me3/H3K27Ac, versus the repressive histone mark H3K9me3/H3K27me3. In addition, we identified that inhibition of SOX2‐OT and reduced expression of SOX2/GLI‐1 sensitizes lung cancer cells to EGFR/TKI‐erlotinib or cisplatin‐based treatment. Finally, we show that high co‐expression of SOX2‐OT/SOX2 transcripts and SOX2/GLI‐1 proteins appears to correlate with a poor clinical prognosis and lung malignant phenotype. Collectively, these results present evidence that LncRNA SOX2‐OT modulates an orchestrated resistance mechanism, promoting poor prognosis and human lung malignancy through genetic, epigenetic, and post‐translational mechanisms.

## Graphical abstract

Molecular model represents pieces of evidence, suggesting that LncRNA SOX2‐OT promotes AKT/ERK phosphorylation, as well as histone mark modifications (Activation H3K4me3/H3K27Ac versus Repression H3K9me3/H3K27me3) at GLI‐1 and SOX2‐OT gene promoter sequences (Likely SOX2) in a probable indirect manner (Dotted lines). Supporting that LncRNA SOX2‐OT is post‐translational and epigenetically involved in therapy resistance mechanisms, lung cancer malignancy, therapy resistance mechanisms, and clinical prognosis.

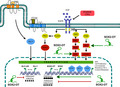

**Abbreviations**
ceRNA: completing endogenous RNA
EGFR: epidermal growth factor receptor
GLI‐1: GLI family zinc finger transcription factor 1
LncRNA: long noncoding RNA
NSCLC: non‐small‐cell lung cancer
RT‐qPCR: quantitative reverse transcription PCR
siRNA: small‐interfering RNA
SOX2: SRY‐Box transcription factor 2
SOX2‐OT: SOX2 overlapping transcript
TKI: tyrosine kinase inhibitors

## 1 Introduction

Lung cancer represents the leading cause of death by malignant diseases, worldwide [1]. Different genomic and genetic alterations pertaining to the hallmarks of cancer determine the initiation, promotion, histopathological transformation, and malignant progression of lung cancer. The role of long noncoding RNAs (LncRNAs) has also been explored, particularly in terms of failure in genetic transcription and/or epigenetic regulation, which have been described as modulated functional archetypes of these molecules [2, 3]. LncRNAs are described as noncodifying RNA molecules greater than 200 nucleotides [4], and several LncRNAs have been known to act as oncogenes, promoting the development and/or progression of lung cancer [5, 6, 7, 8].

LncRNA SOX2 overlapping transcript (SOX2‐OT) is transcribed from two transcription start sites, both in normal embryological development as well as during lung cancer oncogenesis. SOX2‐OT represents a 3.4‐kb‐long transcript, derived from 10 exon units, and produces 6 postsplicing genetic variants. Both SOX2‐OT and, its genome neighbor, transcriptional factor of pluripotency SRY‐Box Transcription Factor (SOX2), are transcribed on the same genetic positive direction [9, 10], suggesting a coordinated genetic co‐expression pattern. Furthermore, experimental evidence supports that LncRNA SOX2‐OT modulates the expression of SOX2 during the embryonic stage as well as during cancer development [11, 12, 13, 14, 15]. LncRNA SOX2‐OT appears to have a relevant role in several malignant diseases including osteosarcoma [16], ovarian cancer [17], gastric cancer [18], hepatocellular carcinoma [19], cholangiocarcinoma [14], esophageal squamous cell carcinomas [20], multiple myeloma [21], bladder cancer [15], and lung cancer [22]. Evidence has shown that SOX2‐OT is involved in mechanisms pertaining to cell cycle regulation, tumor cell proliferation and malignant invasion [22, 23, 24]. Furthermore, SOX2‐OT has been proposed as a ncRNA biomarker for diagnostic and prognostic purposes in patients with malignant lung diseases [25, 26], highlighting that SOX2‐OT is associated with poor patient survival, probably by promoting epigenetic aberrations in lung cancer [22].

However, several important aspects remain unknown, including whether co‐ and overexpression of SOX2‐OT and SOX2 might impact clinically relevant outcomes, including disease progression and response to therapy. The role of SOX2‐OT as a possible epigenetic regulator of the *SOX2* gene promoter has also remained unexplored. Last, the impact at post‐translational level on molecular intermediary members of the EGFR intracellular signaling pathway, as a targeted therapy, has not been described.

The *SOX2* gene has been shown to possess transcriptional ability to regulate the expression of the *GLI‐1* gene [27], participating as a member of the Sonic Hedgehog (Sh) pathway in cancer therapy resistance mechanisms of lung neoplasms [28]. Based on the aforementioned information, we conducted the present study which sought to provide information regarding the role of SOX2‐OT in modulating gene expression of SOX2 as well as its effect in lung cancer therapy resistance and clinical outcomes.

We hypothesized that the SOX2‐OT/SOX2/GLI‐1 molecular axis is involved as a resistance mechanism in lung cancer therapy, and that LncRNA SOX2‐OT acts as an epigenetic regulator of the genetic expression of the SOX2‐OT/SOX2/GLI‐1 genes, involved in resistance mechanisms to lung cancer therapy agents, including cisplatin and EGRF‐tyrosine kinase inhibitor (TKI) erlotinib.

## 2 Materials and methods

### 2.1 Human lung cancer cell lines, cellular cultures, and lung cancer samples

Human wild‐type (*EGFR* and *KRAS* mutation) lung adenocarcinoma cell lines (A549, G12C and NCI‐H2347, G57T) and mutant *EGFR* cell lines (NCI‐H1975, L858R/T790M and HCC827 [delE746‐A750]) were purchased from the American Type Culture Collection (ATCC) (University Boulevard, Manassas, VA, USA). All cells were grown in RPMI‐1640 medium supplemented with 10% fetal bovine serum (Biowest, Riverside, MO, USA), and 1% ampicillin/streptomycin (Biowest). The cells were cultured at 37 °C in a humidified atmosphere containing 5% CO_2_.

Tissue samples from human lung carcinomas were obtained from subjects with advanced non‐small‐cell lung cancer (NSCLC) who attended the Thoracic Oncology Unit at the National Cancer Institute (INCan) in Mexico City from 2005 to 2008. A total of 33 samples were obtained for LncRNA and mRNA expression analysis. Twenty‐three samples (*n* = 23/33) were obtained from patients who had been exposed to known risk factors, including tobacco smoking and/or wood smoke exposure (Risk Smoking Lung Tumors subgroup), while 10 samples (*n* = 10/33) were obtained from subjects without exposure to these risk factors (Nonrisk Smoking Lung Tumors subgroup). Last three normal, nonmalignant, lung tissue samples were obtained for comparative purposes.

Another set of tissue samples (40 lung carcinomas and 25 nonmalignant normal lung tissue) were obtained for the protein co‐expression analyses. The LncRNA SOX2‐OT expression was performed using an additional cohort of lung carcinoma tissue samples from 16 patients, and an additional five normal, nonmalignant lung tissue samples for comparative purposes. These last samples were obtained from the National Institute of Respiratory Diseases (INER), Ismael Cosío Villegas. All experiments using human specimens were carried out in accordance with the Declaration of Helsinki, and were approved by the Institutional Review Committees for INER (B17‐07, B09‐08, C61‐16), and INCAN (008102510M1, CB451). All patients provided written informed consent for the surgical resection of the tissue sample and for its future use in genetic/epigenetic research studies.

### 2.2 Oncological treatment

The EGFR‐TKI‐erlotinib and cisplatin were purchased from Sigma‐Aldrich (Toluca, Mexico). Erlotinib was dissolved in DMSO, the final concentration of DMSO was 2.5% in medium. Cisplatin was dissolved in medium culture. Human lung adenocarcinoma cells were seeded in 10 cm dishes to attach overnight. Briefly we obtained the IC25 of TKI‐erlotinib (TKI‐ER) and cisplatin (CisPlat) for each cell line. The next day, cells were treated with the IC25 of each drug according to cell line (A549: TKI‐ER: 10.10 µm, CisPlat: 2.76 µm, NCI‐H2347: TKI‐ER: 5.16 µm, CisPlat: 1.267 µm, NCI‐H1975: TKI‐ER: 14.785 µm, CisPlat: 1.735 µm and HCC827: TKI‐ER: 15 µm, CisPlat: 1.152 µm). Cells were harvested for futures assays after 12, 48, and 120 h of incubation.

### 2.3 RNA isolation and RT‐qPCR assays

Total RNA was isolated from parental human lung adenocarcinoma cell lines, cell cultures with different treatment conditions and human lung cancer tissue samples, using TRIzol (Invitrogen, Carlsbad, CA, USA) according to the manufacturer's instructions. The concentration for RNA was evaluated by spectrophotometry using an UV/VIS spectrophotometer (Implen NanoPhotometer^™^ NP80, Thermo Fisher Scientific, Madrid, Spain) . One microgram of total RNA was used for complementary DNA (cDNA) synthesis with random hexamers using RevertAid H Minus First Strand cDNA synthesis Kit (Thermo Scientific, Waltham, MA, USA). Mix‐Control was used for baseline expression for cancer cell lines in baseline conditions. The mix‐Control contained equimolar amounts of total RNA from cancer cell lines. The expression profiles of LncRNAs and mRNAs were determined by quantitative PCR using SYBR Green (RealQ Plus Master Mix; AMPLIQON, Odense M, Denmark), with U1 as internal control for LncRNAs and GAPDH as internal control for mRNAs. The PCR primers used in this study are listed in Table S1. The conditions for qPCR were as follows: initial denaturation at 95 °C for 15 min, 40 cycles of amplification: 95 °C for 30 s, 60 °C for 1 min. Melt curve: 95 °C for 5 min, 65 °C for 1 min, and 97 °C continuous. The qPCR was carried out using the LightCycler 480 System Real‐TiME PCR instrument (Roche, Mannheim, Germany). Differences in relative expression were calculated using the $2^{‐\Delta \Delta C_{t}}$ or $2^{‐\Delta C_{t}}$ method. All reactions were performed in experimental triplicates.

### 2.4 Transient transfection (siRNA)

For transfection assays, 3 × 10^5^ A549 (wild‐type) and NCI‐H1975 (*EGFR‐*mutated) lung cancer cells were plated in 6‐well plates overnight and transfected with 50 ng of SOX2‐OT siRNA (siOT1 and siOT2) and equimolar amounts of siRNA siOT1/siOT2 (siMix) using Lipofectamine 2000 (Invitrogen) according to the manufacturer's protocol. The target sequence for SOX2‐OT siRNA has been previously reported [22]. Nontargeting SOX2‐OT RNA siRNA was used as a control (Control siRNA‐A/sc‐37007; Santa Cruz Biotechnology, Dallas, TX, USA). Sequences are provided in Table S2. Cells were harvested 48 h after transfection for future assays.

### 2.5 Western blot analysis

Cell cultures with different treatment conditions were lysed in RIPA buffer (HEPES‐KOH 1 m pH 7.5, NaCl 5 m, EDTA 0.5 m pH 8, Triton X‐100 20%, Sodium Deoxycholate 10%, SDS 20%) containing proteinase inhibitors (complete Mini, Roche, Indianapolis, IN, USA) and phosphatase inhibitors (Sigma Aldrich). Protein concentrations were determined using Bio‐Rad DC™ Protein Assay kit (Bio‐Rad, Hercules, CA, USA), and absorbance was determined by a multiwell spectrophotometer (Epoch, BioTek Instruments, Winooski, VT, USA), at 750 nm. For western blot analysis, 30 µg of protein was separated by 10% SDS/PAGE gel and transferred to a PVDF membrane (Bio‐Rad). Blocking was performed at room temperature for 2 h in TBS‐T with 5% milk (Blotting‐Grade Blocker; Bio‐Rad), followed by overnight incubation with different primary antibodies at 4 °C. Primary antibodies against the following proteins and dilutions were used: GLI‐1: 1 : 500 (Abcam, Cambridge Science Park, Cambridge, UK), SOX2: 1 : 2000 (GeneTex, Irvine, CA, USA), GAPDH: 1 : 3000 (Santa Cruz, Dallas, TX, USA), p‐AKT: 1 : 500, t‐AKT: 1 : 500 (Cell Signaling Technology, Beverly, MA, USA), p‐ERK: 1 : 500, and t‐ERK: 1 : 500 (Cell Signaling Technology). After primary antibody incubation overnight, washing and incubation with secondary antibodies was performed: anti‐mouse or anti‐rabbit (Cell Signaling Technology), at 1 : 10 000 dilution. Proteins were visualized using an enhanced chemiluminescence detection reagent (Clarity™ Western ECL Substrate; Bio‐Rad). GAPDH was used as loading control. The intensities of protein levels were analyzed using imagej software (Research Services Branch, National Institute of Mental Health, Bethesda, MD, USA).

### 2.6 Cellular viability (MTS assays)

EGFR‐TKI‐erlotinib (Sigma‐Aldrich) was dissolved in DMSO as stock solution at 10 mm. Cisplatin (Sigma‐Aldrich) was dissolved in NaCI 0.9% as stock solution at 5 mm. Briefly, A549 and NCI‐H1975 cells transfected with SOX2‐OT siRNA (4 × 10^3^ cells/well for quadruplicate) were seeded in 96‐well plates in RPMI‐1640 containing 10% FBS to attach overnight, then cells were treated with drugs in different serial dilutions (0, 0.01, 0.1, 1, 10, 100, 1000 µm). Control cells for the erlotinib‐treated group were incubated in complete medium with DMSO, the final concentration of DMSO was 0.1–2.5% in medium. Control cells for the cisplatin‐treated group were incubated in complete medium. After 72 h of incubation in the case of erlotinib, and 48 h in the case of cisplatin, 20 µL of MTS CellTiter 96 Aqueous One Solution Cell Proliferation Assay (Promega, Madison, WI, USA) was added and incubated at 37 °C for 2 h. Absorbance was determined by a multiwell spectrophotometer (Epoch, BioTek Instruments), at 490 and 690 nm for background. The inhibitory concentrations (IC50, IC25 and IC10) were calculated by graphpad prism software 6.0 (GraphPad Software, San Diego, CA, USA).

### 2.7 Clonogenic assays

Briefly, A549 cells seeded in 6‐well plates at 3 × 10^5^ cells/dish were transfected by siSCR or siMix (siOT1/siOT2); 24 h later, transfected cells were harvested and seeded in 12‐well plates by triplicate (5 × 10^3^ cells/dish). Cells were left to adhere overnight at 37 °C. After 48 h, cells were exposed to TKI‐Erlotinib (IC25: 9.5 µm) or cisplatin (IC25: 0.75 µm) each 72 h for 21 days. Pharmacological drugs were removed, and cells were washed with 1× PBS and then incubated by drug medium. Cellular colonies were fixed by methanol and acetic acid (3 : 1 v/v) for 5 min, solution was removed and plates were washed by running water. Cells were stained with 0.5% crystal violet for 5 min. Cellular colony growth was quantified using the imagej Colony Number plugin tools software.

### 2.8 Chromatin immunoprecipitation (ChIP) and ChIP‐qPCR assays

Briefly, A549 cells were seeded in 6‐well plates at 3 × 10^5^ cells/dish, and transfected using siSCR and siMix (siOT1/siOT2) sequences; 48 h after transfection, they were cross‐linked with 1% formaldehyde for 10 min, and immediately neutralized with 0.125 m glycine for 5 min. The resulting cells were washed with 1× PBS and treated with lysis buffer (50 mm HEPES‐KOH pH 7.5, 140 mm NaCl, 1 mm EDTA pH 8, 1% Triton X‐100, 0.1% sodium deoxycholate, and 0.1% SDS) containing protease inhibitors (complete Mini; Roche, Indianapolis, IN, USA). The chromatin was sonicated for 20 pulses of 20 s w/u 60 watts. Then, 20 μg of fragmented chromatin was immunoprecipitated (ChIP) using a commercial EZ‐Magna ChIPTM G kit (Millipore, Temecula, CA, USA), and 2 μg of anti‐MEOX2 (Santa Cruz Biotechnology, Dallas, TX, USA), as well as, 1 μg of activated anti‐H3K27Ac, anti‐H3K4me3, anti‐H3K27me3, anti‐H3K9me3 antibodies were added (Abcam). One microgram of anti‐mouse IgG was used as a negative control for ChIP (Millipore, Temecula, CA, USA). The IP‐DNAs were analyzed by performing absolute quantification by qPCR using a LightCycler 480 System Real‐TiME PCR instrument (Roche, Mannheim, Germany), and RealQ Plus 2× Master Mix (AMPLIQON). A total of 20 ng of IP‐DNA was used per reaction, followed by the amplification of standard curves through serial dilutions of native DNA (diploid control genomic DNA) derived from peripheral blood mononuclear cells of healthy donors (100, 10, 1.0, 0.1, and 0.01 ng). The following amplification conditions were used: initial denaturation at 95 °C for 10 min and 40 cycles of denaturation at 95 °C for 15 s, annealing at 55 °C for 30 s and extension at 72 °C for 30 s. All oligonucleotides set for the proposed gene promoter target sequences are listed in Table S3 and were designed using the Primer3Plus program and synthetized by T4OLIGO Company (Irapuato, Mexico).

### 2.9 Immunofluorescence analysis

Samples were incubated with primary anti‐GLI‐1 (Abcam; ab49314) and anti‐SOX2 (Santa Cruz; Sc‐365823) antibodies in PBS and 1% BSA overnight. Samples were washed twice by PBS and incubated with secondary antibodies: anti‐mouse CFL488 (Santa Cruz; Sc‐395759) and anti‐rabbit CFL647 (Santa Cruz; Sc‐3739) in PBS and 1% BSA for 45 min followed by three washes with 0.1% PBS‐Tween, incubation with 2 ng·mL^−1^ DAPI, three washes with 0.1% PBS‐Tween, and two washes with PBS. Images were acquired in a fluorescence microscope, AXIO LAB1 from ®ZEISS (Jena, Germany). Representative pictures were taken at 100× magnification. Imaging analysis was performed with imagej software 1.8.0.

### 2.10 Statistical analysis

graphpad prism 6 (GraphPad Software, Inc.) was used for statistical analyses. All data were represented as mean ± standard deviation (SD) of three independent experiments. Differences between two groups were assessed using unpaired *t*‐test (two‐tailed). For normally distributed data with unequal variance, an unpaired *t*‐test with Welch's correction was used. The spss (IBM SPSS Statistics version 15, Chicago, IL, USA) software was used for the statistical analysis of clinical variables in lung cancer patients. The chi‐square test was used to assess differences in the baseline characteristics of the population. Univariate analysis was used to analyze clinical and oncological variables of lung cancer patients associated with differential expression of target genes. Kaplan–Meier overall survival analyses were performed according to the relative SOX2‐OT, SOX2, GLI1‐AS, and GLI‐1 expression level, survival curves were examined using the log‐rank test. Significance was defined as *P < 0.05, **P < 0.01; ***P < 0.001, and ****P < 0.0001.

## 3 Results

### 3.1 Co‐expressed LncRNA‐SOX2‐OT/mRNA‐SOX2 binomial by lung cancer cells *in vitro*

**Fig. 1:**
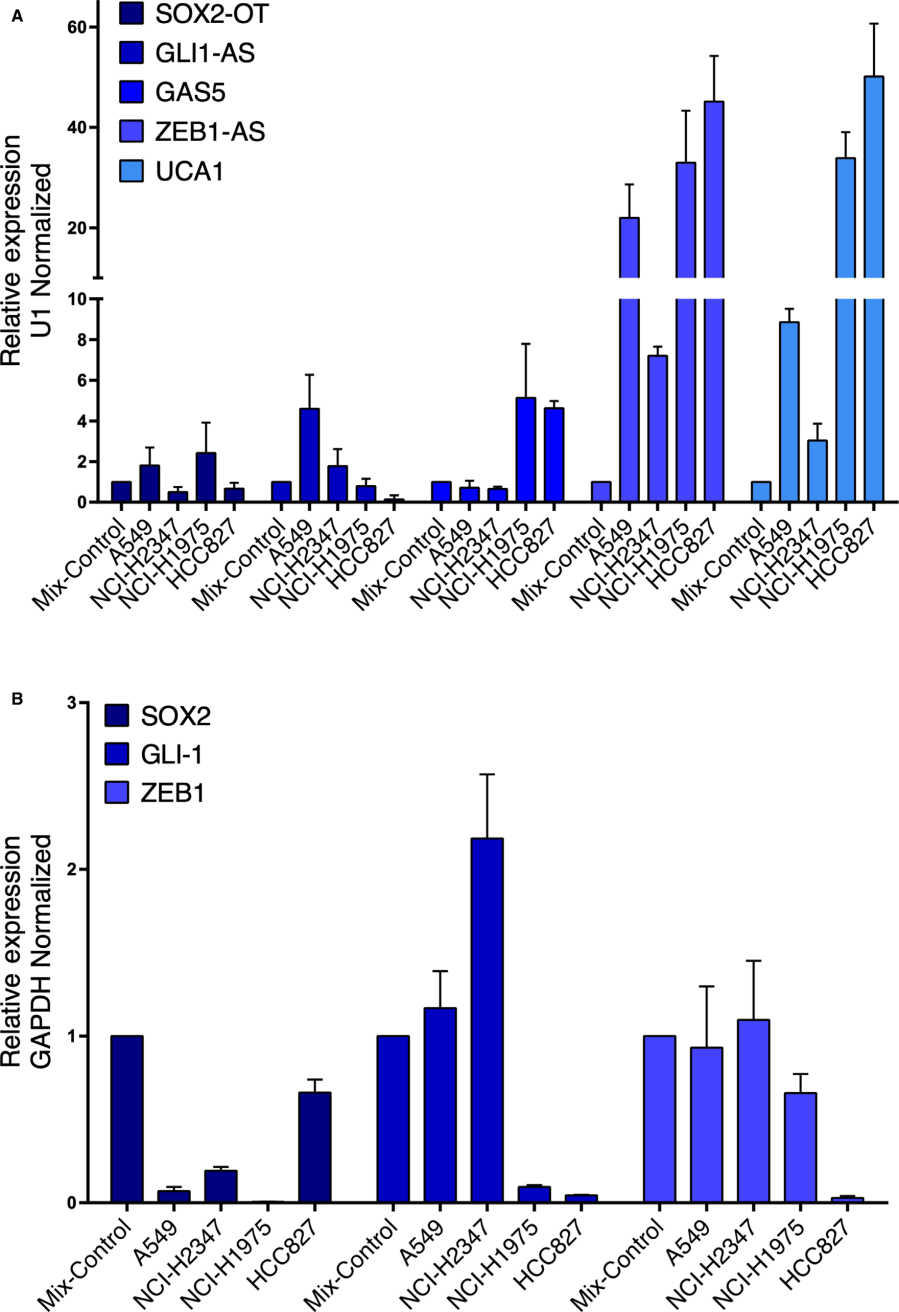
Analyses of the genetic expression pattern of LncRNAs, and LncRNA‐mRNA binomial axes, at baseline cell culture conditions in human lung cancer cell lines. (A) LncRNAs relative expression profile in four human lung cancer lines, and (B) mRNAs coding genes expression profile in four lung cancer cell lines, by qPCR assays. Error bars represent the mean ± SD of three independent experiments. Mix‐Control is a pool of equal amounts of each total RNA in lung cancer cell lines.

Differential expression patterns and levels of the LncRNA SOX2‐OT, in contrast to other LncRNAs (GLI1‐AS, GAS5, ZEB1‐AS and UCA1), were identified in several lung cancer cell lines at baseline cellular culture conditions. The highest expression levels for ZEB1‐AS and UCA1 were identified on A549, NCI‐H2347, NCI‐H1975 and HCC827 lung cancer cells (Fig. 1A). In contrast, both *EGFR*‐mutated lung cancer cells NCI‐H1975 and HCC827 had higher expression levels for LncRNA GAS5. However, it was possible to detect overexpressed GLI1‐AS in both *EGFR*‐wild‐type lung cancer cells A549 and NCI‐H2347. Overexpression of SOX2‐OT levels were mainly detected in A549 (*EGFR*‐wild‐type) and NCI‐H1975 (*EGFR*‐mutated) cells (Fig. 1A). Additionally, in baseline cell culture conditions, we observed a probable negative correlation in co‐expression of the LncRNA SOX2‐OT with mRNA SOX2; a similar pattern was identified for the LncRNA ZEB1‐AS with its mRNA ZEB1 (Fig. 1A,B). Whereas a positive correlation in the co‐expressed LncRNA GLI1‐AS with mRNA GLI‐1, were detected (Fig. 1A,B). Based on this data, the possible co‐induced phenomenon of the LncRNAs, with its respective neighborhood mRNA, by oncological‐drug treatments was analyzed.

### 3.2 Co‐expressed SOX2‐OT/SOX2 binomial is positively induced by cisplatin or EGFR‐TKI treatment in lung cancer cells

**Fig. 2:**
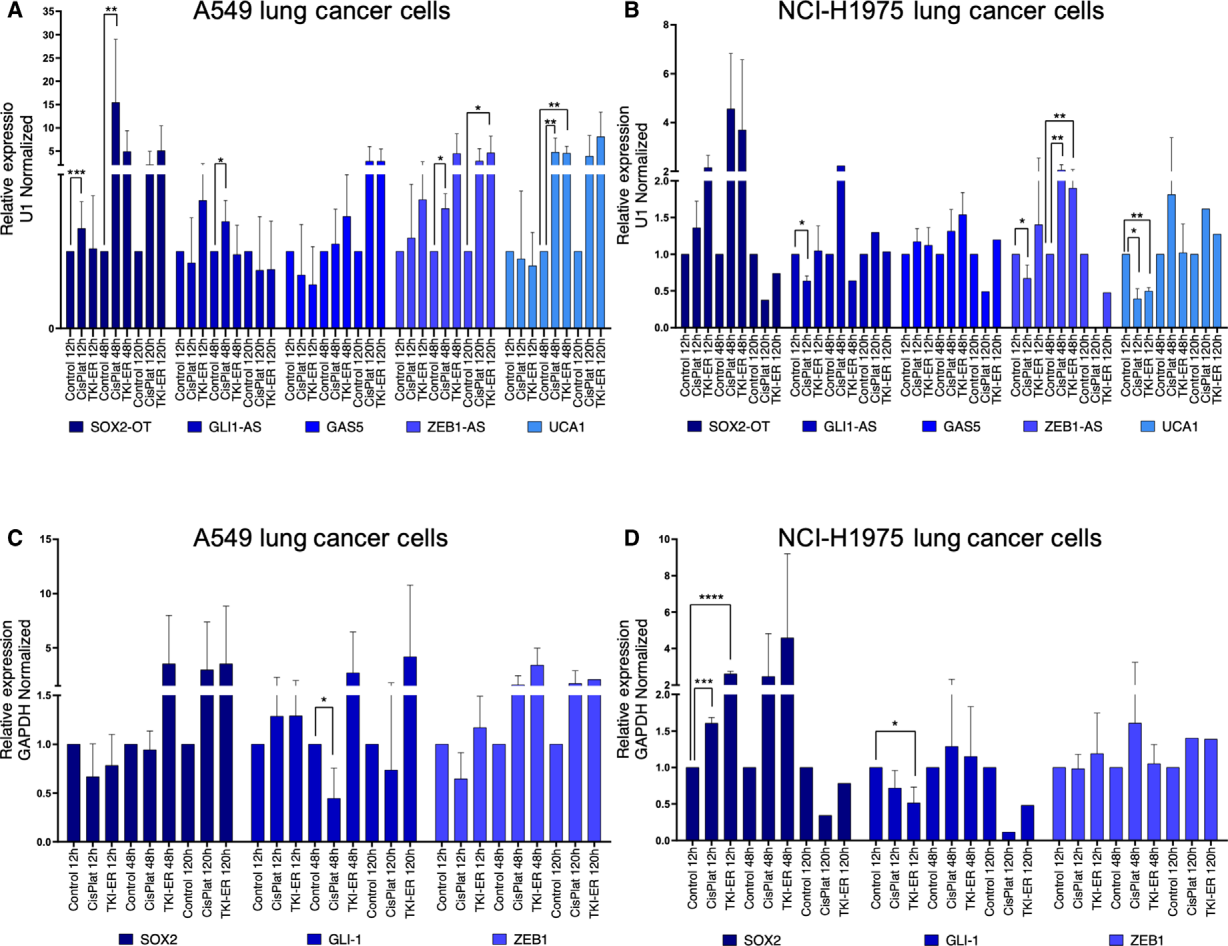
Analyses of the induced relative gene expression, in the presence of the cisplatin or EGFR‐TKI‐Erlotinib treatment in *EGFR*‐wild‐type and *EGFR*‐mutated lung cancer cells. (A) LncRNA expression pattern analyses on EGFR‐wild‐type A549 cells, and (B) *EGFR*‐mutated NCI‐H1975 lung cancer cells. (C) mRNA expression profile in A549 cells, and (D) NCI‐H1975 cells. Relative expression levels have been analyzed by RT‐qPCR assays. Error bars represent the mean ± SD of three independent experiments. CisPlat: cisplatin, TKI‐ER: TKI‐erlotinib. Unpaired *t‐*test (Two‐tailed) used to compare to the control and combination groups. *P < 0.05, **P < 0.01, ***P < 0.001, ****P < 0.0001.

Lung cancer cell lines A549 and NCI‐H1975 were stimulated by *in vitro* incubation with either cisplatin (IC25: 2.76 µm and IC25: 1.735 µm, respectively) or TKI‐erlotinib (IC25: 10.10 µm and IC25: 14.785 µm, respectively) for 12, 48 and 120 h. Results showed a significant inducible expression pattern of the LncRNA SOX2‐OT for lung cancer cells A549 (Fig. 2A), and NCI‐H1975 (Fig. 2B), as well as significant changes for both LncRNAs ZEB1‐AS and UCA1, while a nonsignificant inducible expression pattern for both LncRNAs GLI1‐AS and GAS5 was detected in A549 (Fig. 2A), and NCI‐H1975 lung cancer cells (Fig. 2B). Further, we observed an increase in the induced co‐expression pattern of the SOX2‐OT with SOX2 (Fig. 2A) after treatment with cisplatin or TKI‐erlotinib at 48 and 120 h in lung cancer cells A549 (Fig. 2C). An increase in the co‐expressed SOX2‐OT (Fig. 2B) and SOX2 at 12 and 48 h was also detected in NCI‐H1975 lung cancer cells (Fig. 2D). All of this contrasted with the significant induced expression of LncRNAs GLI1‐AS/ZEB1‐AS (Fig. 2B), with their nonsignificant induced expression of their corresponding coding genes GLI‐1 and ZEB1, in both A549 and NCI‐H1975 cells (Fig. 2C,D).

Regarding this last point, we had proposed to identify possible epigenetic histone code changes at 5′ upstream of the SOX2‐OT gene promoter sequences induced by oncological drug treatment on lung cancer cells. We identified nonsignificant enrichment changes in the activation histone code H3K4me3/H3K27Ac, and a slight decrease in repressive histone H3K27me3 (Fig. S1). This would imply that there is an unknown molecular mechanism involved in the inducible pattern exhibited by exposure to oncological treatment (cisplatin/TKI‐erlotinib) for LncRNA SOX2‐OT.

Nevertheless, we identified that, at baseline conditions, SOX2‐OT variant 1 positively correlates, while SOX2‐OT variant 6 negatively correlates, with mRNA SOX2/GLI‐1 expression, in A549 cells, compared with a heterogeneous expression level in different lung cancer cells (Fig. S2A,B). In addition, our analysis identified that relative abundance of SOX2, and GLI‐1 proteins is homogeneously high in A549, NCI‐H2347, and NCI‐H1975, but not in HCC827 lung cancer cells (Fig. S2C). In addition, we have identified high and homogeneous protein levels for total‐AKT and total‐ERK at baseline conditions (Fig. S2C). We also observed that such total‐AKT and ‐ERK protein levels (Fig. S2D), as well as phosphorylated AKT and phosphorylated ERK proteins levels (Fig. S2E), remain homogeneously elevated in the presence of cisplatin or TKI‐erlotinib, at 12, 48 and 120 h, in A549 cells. In consequence to the observation that LncRNA SOX2‐OT is overexpressed following drug exposure, we explored whether total or phosphorylated AKT and ‐ERK protein levels are positive or negatively regulated by SOX2‐OT, in lung cancer cells.

### 3.3 SOX2‐OT positively promotes total‐AKT/ERK and phosphorylated‐AKT protein levels, as well as resistance to oncological therapy

**Fig. 3:**
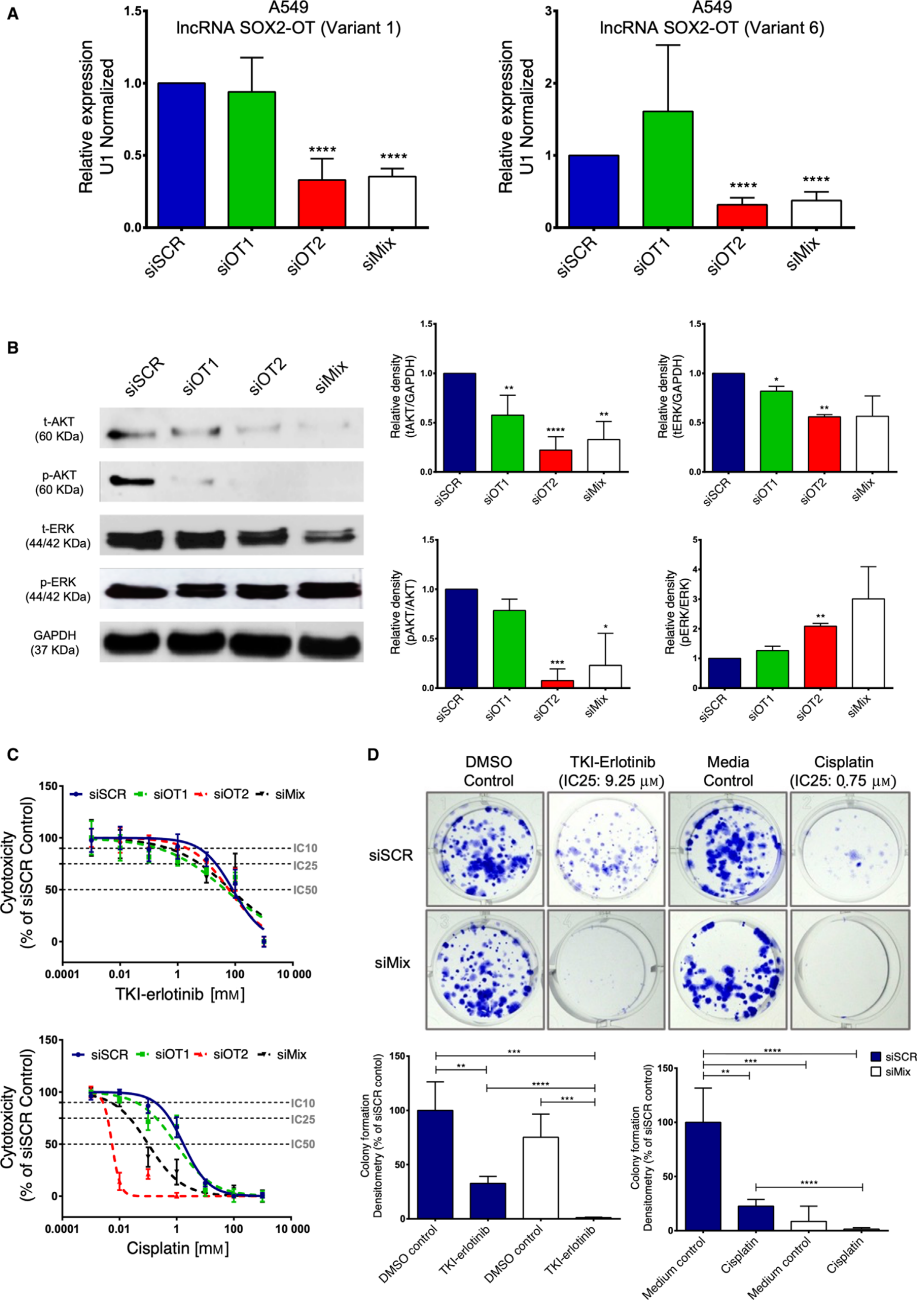
Silenced LncRNA SOX2‐OT promotes reduction in the relative abundance levels of total‐AKT/ERK proteins, as well as, phosphorylated‐AKT/ERK protein levels, and promotes sensitivity to EGFR‐TKI and cisplatin treatment in lung cancer cells. (A) Genetic silencing assays for LncRNA SOX2‐OT variants 1 and 6, and RT‐qPCR assay analysis, identified a reduction in SOX‐OT relative expression level. (B) Total AKT, ERK and phosphorylated‐AKT, ERK levels were obtained by western blot analysis and relative densitometric analysis. (C) Cellular cytotoxicity assays identified inhibitory concentrations (IC) for the TKI‐erlotinib response as follows: siSCR group: IC50 = 82.45 µm, siOT1 group: IC50 = 45.57 µm, siOT2 group: IC50 = 60.26 µm, and siMix group: IC50 = 70.76 µm. siSCR group: IC25 = 21.54 µm, siOT1 group: IC25 = 3.039 µm, siOT2 group: IC25 = 11.25 µm, and siMix group: IC25 = 5.778 µm. siSCR group: IC10 = 5.523 µm, siOT1 group: IC10 = 0.2027 µm, siOT2 group: IC10 = 2.102 µm, and siMix group: IC10 = 0.4719 µm. Cellular cytotoxicity assays identified inhibitory concentrations (IC) for the Cisplatin response as follows: siSCR group IC50 = 1.723 µm, siOT1 group IC50 = 0.952, siOT2 group IC50 = 0.006 µm and siMix group IC50 = 0.101 µm. siSCR group IC25 = 0.553 µm, siOT1 group IC25 = 0.1918 µm, siOT2 group IC25 = 0.004, and siMix group IC25 = 0.022 µm. siSCR group IC10 = 0.179 µm, siOT1 group IC10 = 0.039 µm, siOT2 group IC10 = 0.003 µm, and siMix group IC10 = 0.005 µm. (D) Images from the clonogenic assay for transfected A549 siMix‐SOX2‐OT. Cells were exposed to IC25 = 7.5 µm at erlotinib and IC25 = 0.75 µm at cisplatin for 21 days. IC25 were previously obtained for MTT assays in A549 lung cancer parental cells (data not shown). Error bars represent the mean ± SD of three independent experimental assays. Unpaired *t‐*test (Two‐tailed) was used to compare control (siSCR) group, and experimental combination groups. *P < 0.05, **P < 0.01, ***P < 0.001, ****P < 0.0001.

Next, using genetic silencing (siRNAs) assays we show that LncRNA SOX2‐OT controls the relative abundance of the total and phosphorylated‐AKT and ‐ERK proteins, promoting resistance mechanisms to oncological‐drug treatments. First, both siRNAs, SOX2‐OT variant 1 (siOT1) and 6 (siOT2), reduced SOX2‐OT expression in A549 (Fig. 3A), and NCI‐H1975 lung cancer cells (Fig. S3A). Second, both siOT1 and siOT2 (siRNA‐Mix) significantly reduced total‐AKT and total‐ERK protein levels, as well as decreased phosphorylated‐AKT protein level (Fig. 3B). In contrast, silenced SOX2‐OT promotes increased ERK‐phosphorylated levels (Fig. 3B). Suggesting that activation of the PI3K‐AKT pathway occurs in a SOX2‐OT‐dependent manner, while the activated KRAS‐ERK pathway is only partially dependent on SOX2‐OT. Nevertheless, in NCI‐H1975 lung cancer cells we did not identify any significant changes in the total AKT‐ERK protein levels, but observed an increase in phosphorylated pAKT/pERK protein levels (Fig. S3B). Based on this information, it was possible to determine that SOX2‐OT increases resistance to EGFR‐TKI‐erlotinib or cisplatin treatment in A549 cells (Fig. 3C), as well as resistance to therapy in NCI‐H1975 lung cancer cells (Fig. S3C). To provide additional evidence for the involvement of the LncRNA in regard to therapy resistance, we carried out clonogenic (cellular proliferation) assays through genetic silencing by siMix‐SOX2‐OT on A549 cells. Transfected lung cancer cells were exposed to TKI‐erlotinib or cisplatin using an IC25 pharmacological concentration every 72 h for 21 days. Genetically silenced or reduced SOX2‐OT levels decreased cellular proliferation and promoted sensitivity to both drugs (TKI‐erlotinib/cisplatin) (Fig. 3D). It is likely that SOX2‐OT is involved in transcriptional epigenetic regulation of the *SOX‐GLI‐1* gene axis, and also self‐regulation.

### 3.4 Silenced LncRNA SOX2‐OT reduces co‐expressed SOX2 and GLI‐1 genes, affecting histone code H3K4me3/H3K9me3/H3K27me3 at gene promoter sequences

**Fig. 4:**
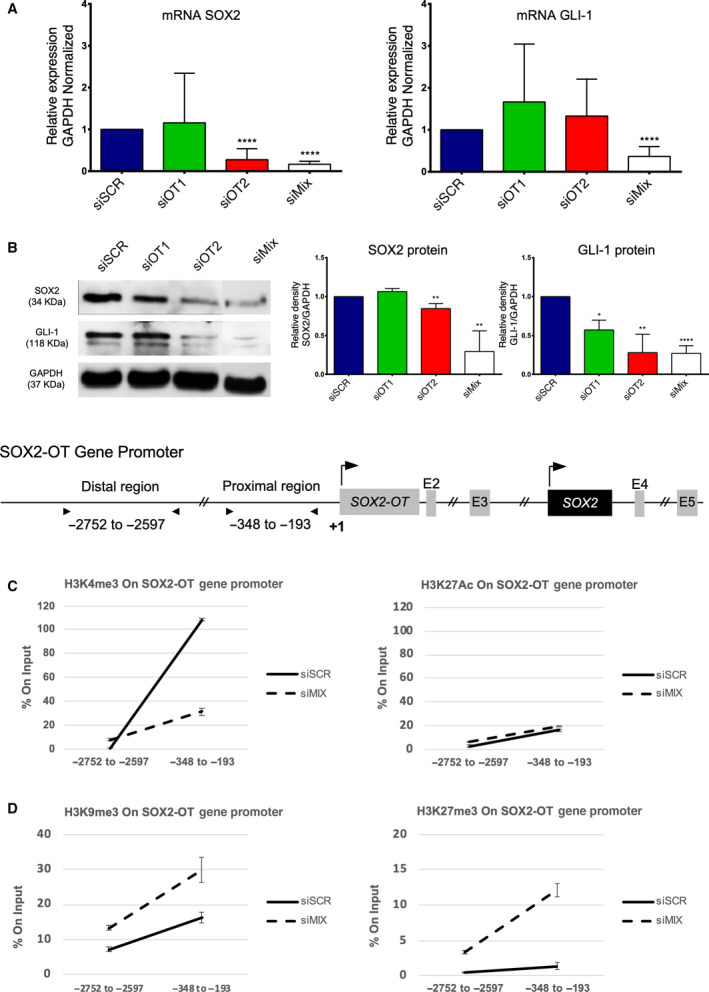
Silenced LncRNA SOX2‐OT promotes a decrease in mRNA transcript expression and SOX2 and GLI‐1 protein level, as well as promotes changes in the histone enrichment of the H3K4me3/H3K9me3/H3K27me3 chromatin profiles at SOX2‐OT gene promoter sequences in lung cancer cells. (A) Genetic silencing assays (by siRNAs anti‐LncRNA SOX2‐OT) of the SOX2‐OT reduced mRNA expression of both SOX2 and GLI‐1. (B) Reduction in the SOX2 and GLI‐1 protein levels caused by siRNA anti‐SOX2‐OT. (C) Map of the SOX2‐OT and SOX2 gene coding and promoter sequences. Reduction in the histone enrichment H3K4me3 and no changes for histone enrichment H3K27Ac. (D) Increasing of the enrichment histone code H3K9me3/H3K27me3 on SOX2‐OT gene promoter sequences ‘distal and proximal regions’. Error bars represent the mean ± SD of three independent experiments. Unpaired *t‐*test (Two‐tailed) used to compare to the control (siSCR) group and experimental combination groups *P < 0.05, **P < 0.01, ****P < 0.0001.

Next, we identified that siMix‐SOX2‐OT positively controls both genetic expression of SOX2‐GLI‐1 mRNAs (Fig. 4A) and relative abundance of SOX2 and GLI‐1 protein levels, in A549 lung cancer cells (Fig. 4B). This specific‐genetic scenario was also identified both in mRNA and protein levels in NCI‐H1975 lung cancer cells (Fig. S3B,D). On the other hand, also using genetic silencing siMix‐SOX2‐OT assays, we identified that LncRNA SOX2‐OT is able to promote a positive epigenetic biofeedback, by the enrichment of activated histone H3K4me3 levels, with no changes of H3K27Ac at the SOX2‐OT gene promoter sequences (Fig. 4C). Interestingly, LncRNA SOX2‐OT significantly reduced repressive histone enrichment, H3K9me3/H3K27me3, at the SOX2‐OT promoter sequences (Fig. 4D). Additionally, we detected that LncRNA SOX2‐OT negatively controls to the enrichment of activated histones H3K4me3/H3K27Ac (Fig. S4A), but slightly controls the repressive histones H3K9me3/H3K27me3 levels at GLI‐1 gene promotor sequences (Fig. S4B). Nevertheless, such epigenetic changes do not explain the reduction in the GLI‐1 mRNA/protein expression. The overexpressed SOX2‐OT/SOX2/GLI‐1 trinomial axis could represent a new molecular signature involved in lung cancer biology and therapy response.

### 3.5 Overexpression of the SOX2‐OT/SOX2/GLI‐1 trinomial axis is associated with clinical outcomes in lung cancer patients

We have identified that significantly overexpressed LncRNA SOX2‐OT levels in human lung carcinomas (Fig. S5A) are associated to poorer overall survival in lung cancer patients, with lower clinical response to oncological treatments (Cisplatin/Taxanes/EGFR‐TKIs), ranging from 1 to 11 months (Table 1). In contrast, down‐expressed SOX2‐OT in human lung carcinomas (Fig. S5A), is associated to better overall survival, ranging from 11 to 94 months, with favorable response to oncological therapy (Table 1). Interestingly, when evaluating normal adjacent lung tissue, we also identified down‐expressed SOX2‐OT levels (Fig. S5A).

**Table 1:** Clinical outcomes for lung cancer patients. TNM, classification of malignant tumors; N.D, not determined; AD, adenocarcinoma.

Patient	Age	Sex	NSCLC type	TNM	Family history of cancer	Follow‐up	Treatment	Clinical response	Current status	LncRNA SOX2‐OT relative expression level	First‐line therapeutic scheme/response
PMA	60	Female	AD	2,3,1	Positive	94 Months	Adjuvant chemotherapy/Radiotherapy	Partial response, Local dissemination	Alive	Low	CISPLATIN/VINORELBINE partial response
SPN	41	Female	AD	3,2,0	Positive	74 months	Co‐adjuvant and adjuvant chemotherapy/Radiotherapy	Partial response	Alive	Low	CDDP/NAVELBINE partial response
VSB	67	Female	AD	1,2,1	Positive	48 months	Adjuvant chemotherapy	No response, Local and distal dissemination	Deceased	Low	CARBOPLATIN/DOCETAXEL/BEVACIZUMAB partial response
AEE	70	Female	AD	4,0,0	Negative	16 months	Adjuvant chemotherapy/Radiotherapy	Partial response, Local and distal dissemination	Deceased	Low	CARBOPLATIN/GEMCITABINE no response
GMRM	48	Female	AD	4,1,1	Negative	13 months	Adjuvant chemotherapy	Partial response	Deceased	Low	CISPLATIN/DOCETAXEL partial response
DSE	71	Female	AD	4,3,1	Negative	11 months	Adjuvant chemotherapy	No response	Deceased	Low	CARBOPLATIN/DOCETAXEL partial response
RSCL	36	Female	AD	4,2,1	Negative	11 months	Adjuvant chemotherapy	No response, Local and distal dissemination	Deceased	High	DOCETAXEL/CISPLATIN partial response
GGR	68	Female	AD	4,0,0	Positive	7 months	Adjuvant chemotherapy/Radiotherapy	N.D.	Deceased	Low	CARBOPLATIN/PACLITAXEL partial response
MGGR	55	Female	AD	N.D.	Negative	6 months	N.D.	N.D.	Deceased	High	N.D.
GCH	74	Female	AD	3,2,1	Negative	1 month	Patient refused treatment	N.D.	Deceased	High	N.D.

**Fig. 5:**
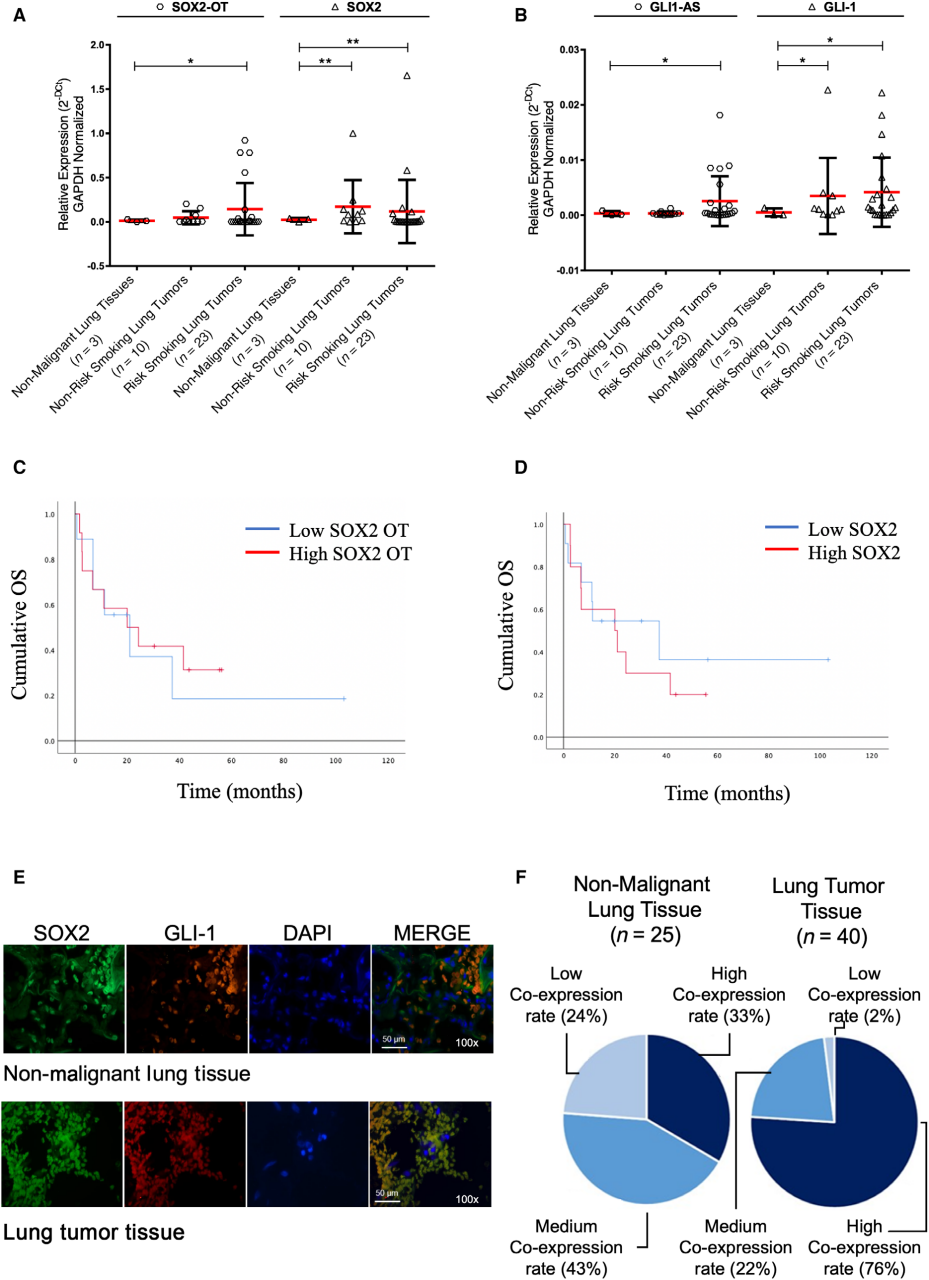
Overexpressed LncRNA SOX2‐OT is associated with a poor prognosis in lung cancer patients independent of risk‐factor exposure, where co‐expressed SOX2‐OT/SOX2/GLI‐1 is associated with a lung malignant phenotype. (A) Relative expression of the co‐expressed pattern LncRNA SOX2‐OT and mRNA SOX2. (B) Relative expression of the co‐expressed LncRNA GLI1‐AS and mRNA GLI‐1, in human lung cancer patients (INCAN Cohort), without risk factors (*n* = 10) and with exposure to risk factors (Tobacco Smoking and/or Wood Smoke‐Risk; *n* = 23), compared with nonmalignant lung tissues (*n* = 3). (C, D) Kaplan–Meier survival analysis for lung carcinoma patients (INCan cohort) with low or high SOX2‐OT and SOX2 expression. Cumulative overall survival (OS) rate was calculated using the log‐rank test. (E) Representative image of co‐expression of SOX2 (green staining) and GLI‐1 (red staining) proteins in human normal lung adjacent tissues and lung tumor tissues (INER Cohort), nuclei were detected by DAPI dye. Co‐localization analysis (merge) was performed by fluorescence microscopy, using 100× magnification. (F) Correlative co‐expression indexes for *SOX2* and *GLI‐1* genes, were determined by higher co‐expression rates in histological samples, associated with a histological malignant phenotype of the human lung carcinoma tissues. A highest co‐expression rate GLI‐1/SOX2 was reached in 33% for the normal lung adjacent tumor tissues (*n* = 25), compared with lung carcinoma tissues (*n* = 40) which had a 76% (higher co‐expression). Scale bar, shown 50 µm. Error bars represent the mean ± SD. Unpaired *t*‐test and Welch's correction, *P < 0.05, **P < 0.01.

In addition, the overexpressed pattern of the co‐expressed SOX2‐OT/SOX2 binomial transcript axis was significantly higher (*P ≤ *0.01) in risk‐factor exposed lung cancer tissue samples compared with nonmalignant lung tissue (Fig. 5A). A significant (*P ≤ *0.05) increase in GLI1‐AS/GLI‐1 binomial expression level was also identified in risk‐factor exposed lung cancer patients (Fig. 5B) compared with normal lung tissue. It is important to highlight that both SOX2 (*P ≤ *0.01), and GLI‐1 (*P ≤ *0.05) genes remain significantly overexpressed in both non‐ and risk‐exposed lung cancer patient subgroups (Fig. 5A,B).

In addition, we evaluated overall survival in patients included in this study and identified that patients with a higher expression of SOX2 reach a median overall survival of 19.97 months (95% CI: 0.00–41.76), while patients with lower SOX2 expression level reached a median overall survival of 37.25 months (95% CI: 3.52–70.99). This contrasts with SOX‐OT expression levels, in which case we did not identify any significant differences in terms of higher or lower expression (20.92 versus 19.97 months median overall survival) (Fig. 5C,D), similar data was obtained pertaining to GLI1‐AS/GLI‐1 expression (Fig. S5B,C).

Furthermore, univariate analyses on clinical outcome of the lung cancer patients let us to identify that oncological metastasis is significatively (*P* ≤ 0.009) associated with overexpressed LncRNA SOX2‐OT levels (Table S4), as well as, overexpressed SOX2 levels significatively (*P* ≤ 0.021) associated with smoking exposition (Table S5). On the other hand, overexpressed GLI1‐AS/GLI‐1 levels, associated with wood smoke exposure (*P* ≤ 0.08) and advanced clinical stages IIIB‐IV (*P* ≤ 0.038) (see Tables S6 and S7).

Finally, *in situ* immunofluorescence analyses identified lower co‐expressed SOX2/GLI‐1 proteins in nonmalignant lung tissue samples (Fig. 5E) with a low co‐expression (24%), medium co‐expression (43%) and higher co‐expression (33%) pattern (Fig. 5F). In contrast, human lung carcinomas showed a low co‐expression (2%), medium co‐expression (22%), and higher co‐expression (76%) SOX2/GLI‐1 protein pattern. This is compatible with a poorer histological differentiation phenotype in advanced clinical stages IIIB‐IV (Fig. 5F). Altogether, the evidence highlights the conclusive role of the SOX2‐OT/SOX2/GLI‐1 trinomial co‐expressed axis in poorer oncological treatment response, and poorer overall survival in lung cancer.

## 4 Discussion

Expression profiles of LncRNAs in different cancer types have been widely reported [29]. In the present work, we identified a significant baseline or inducible expression pattern of several LncRNAs in lung cancer cells, including SOX2‐OT, GLI1‐AS, GAS5, ZEB1‐AS and UCA1, some of which play a key biological role in embryo development and malignant disease progression, including lung cancer [30, 31, 32]. Based on its neighborhood genomic location, binomial‐transcripts (LncRNAs/mRNAs) were identified in a co‐expressed pattern, highlighting that SOX2‐OT/SOX2, and GLI1‐AS/GLI‐1 in human lung cancer cells and solid lung carcinomas probably engage in a dynamic positive/negative correlation, as has been previously reported for prostate and breast carcinomas [33, 34]. Similar findings have been reported in laryngeal squamous cell carcinomas for other binomial LncRNA/mRNA transcripts, such as: NR_003919/PIK3R1, NR_027340/ITGB1, MIR31HG/HIF1A, and SOX2‐OT/DDIT4 [35]. In addition, we detected significant changes in the baseline expression levels for binomial transcript SOX2‐OT/SOX2. A positive correlation had been previously reported in different kind of cancers, including breast, bladder, osteosarcoma, and relevant to this study, lung carcinomas [11, 15, 16, 22].

We have demonstrated using *in vitro* cisplatin or TKI‐erlotinib treatment, routinely used in human lung cancer therapy schemes [36, 37], a significant induced overexpressed LncRNA SOX2‐OT level, in contrast with other LncRNAs, such as GLI1‐AS, GAS5, ZEB1‐AS, and UCA1. Also, overexpressed pluripotency transcription factor *SOX2* and Sonic Hedgehog (Sh) pathway *GLI‐1* effector genes were detected in a pharmacologically induced dependent manner in both A549 and NCI‐H1975 lung cancer cells. It has been previously reported that overexpressed GLI‐1 is associated with cisplatin resistance [28], the co‐expressed GLI‐1/SOX2 transcript axis is related to TKI‐erlotinib [27], and taxane‐gemcitabine‐drug cancer resistance in prostate and lung cancer [38]. Nonetheless, our data shows that a co‐expressed pattern of the SOX2‐OT/SOX2/GLI‐1 ‘trinomial axis’ is likely involved in therapy failure and poorer clinical prognosis, through epigenetic mechanisms which interact in lung cancer drug resistance.

However, our results suggest that cancer drug‐induced LncRNA SOX2‐OT was not the sole result of the activated histone profile H3K4me3/H3K27Ac/RNA Pol II, as these changes were not statistically significant. Nor by a reduction in the repressive histone H3K27me3 on SOX2‐OT gene promoter sequences. Pertaining to this last observation, few reports have detected pharmacological effects—cisplatin or TKIs—on aberrant epigenetic mechanisms, as it has been reported for H3K27me3, or chromatin remodeling protein complexes ‘chromatin‐writers’ levels, in human cancer cells [39]. However, scarce experimental evidence allows us to explain the expression of the SOX2‐OT/SOX2 axis in cancer, which has been recently reported to be genetic/epigenetically regulated through MTA3 and GATA3, probably in a NurD (chromatin remodeling complex) interaction, affecting cancer stemness and metastasis. This would propose the MTA3/SOX2‐OT/SOX2 axis as a potential cancer stratification molecular‐marker, and therapeutic targets in human esophageal squamous cell carcinomas [40]. Additionally, another LncRNA/mRNA binomial axis (GLI1‐AS/GLI‐1) has been reported to be epigenetically regulated (enriched histone H3K27me3) at bidirectional gene promoter sequences, involved in tumor cellular proliferation and *in vivo* xenograft tumor growth [34].

Interestingly, resistance mechanisms to oncological drugs based on drug‐efflux, cell apoptosis, autophagy, and cancer cell stemness, as well as, mutations in TK receptors (e.g. EGFR) have partially been explained for both chemotherapy and targeted therapy in lung cancer [41, 42]. Likewise, resistance mechanisms to cisplatin and TKI‐erlotinib have been associated to increased phosphorylation of the EGFR‐downstream cell signaling molecules, as it occurs for phosphorylated‐AKT and phosphorylated‐ERK intermediary proteins [43, 44]. Based on this, a *KRAS* gene mutation in A549 *EGFR*‐wild‐type lung cancer cells actively promotes over‐phosphorylated EGFR‐AKT levels, keeping its over‐activated level even when using EGFR‐TKIs treatment [45]. Our results propose that LncRNA/SOX2‐OT can disrupt lung cancer therapy resistance capacity, by downregulation of the EGFR‐AKT‐ERK expression and activation rate.

In human lung oncology, poor clinical prognosis (overall survival) has largely been dependent of tumor therapy resistance mechanism capacities [46]. In this regard, the role of LncRNAs as molecular resistance mechanisms to cisplatin/EGFR‐TKIs‐based therapies has recently been reviewed, with discussions focusing on their therapeutic potential in lung cancer [47]. Some LncRNAs including HOTAIR, MALAT1, NEAT1, and NNT‐AS1 have been involved in cisplatin resistance, through WNT and/or MAPK/Slug intracellular signaling pathways, as well as promoting malignancy in solid lung tumors, and lung cancer cell lines [48, 49, 50, 51]. Meanwhile some LncRNAs, as UCA1 and BC087858, promote activation of the EGFR cell signaling pathway members, such as AKT/mTOR, and/or ERK in NSCLC [52, 53]. In this present work, we have highlighted an induced overexpressed SOX2‐OT level, where siRNAs knockdown of SOX2‐OT reduces total‐ and phosphorylated‐AKT protein levels in *EGFR*‐wild‐type, *KRAS*‐mutated A549 lung cancer cells. However, we detected little variation in the phosphorylated‐AKT and phosphorylated‐ERK levels by siRNAs anti‐SOX2‐OT in NCI‐H1975 cells. Therefore, our results support the hypothesis that LncRNAs are functional participants in therapy resistance/sensitivity to cancer drugs, and target‐gene therapies ‘EGFR/AKT/ERK/mTOR’ [47]. It has been previously reported that SOX2‐OT participates in the activation of the AKT‐member of cellular signaling pathways in cholangiocarcinoma [14], as well as being involved in the magnitude of resistance mechanism to EGFR‐TKIs‐based treatment related to the functionality of phosphatase PTEN, reducing AKT phosphorylation in NSCLC [54]. In this sense, PTEN has been previously considered as a tumor suppressor gene, associated with cisplatin resistance mechanisms in gastric cancer [55]. Meanwhile, LncRNAs such as GAS5, TP53TG1, and AC078883.3 have been associated with cisplatin sensitivity through miR‐21/PTEN, miR‐18a/PTEN, and PTEN/AKT axis, respectively, in human lung carcinomas [56, 57, 58]. It has been proposed that overexpression of LncRNA GAS5 might reverse resistance to gefitinib in A549 lung cells, promoting a reduction in p‐AKT, p‐ERK, p‐EGFR, and p‐IGF1R levels through an unknown mechanism [59]. In contrast, our results show that genetic silencing of LncRNA SOX2/OT reduced t‐AKT, t‐ERK, and p‐AKT levels in lung cancer cells.

Based on the beforementioned information, it has been described that the expression of the PTEN phosphatase is epigenetically controlled, through promoter methylation in human lung carcinomas [60]. Tai *et al*. [61] have demonstrated that expression of the PTEN gene is epigenetically controlled by SOX2‐OT, through the recruitment of the enzyme histone methyltransferase EZH2, increasing repressive histone mark H3K27me3 at the PTEN promoter level in epithelial tumors, probably supporting that PTEN targeted‐AKT, epigenetically regulated by SOX2‐OT, is involved in target therapy resistance in epithelial lung tumors.

In addition, due to the intronic location of SOX2‐OT at the SOX2 genetic locus, some studies have described a co‐expression pattern for SOX2‐OT and SOX2 in embryo development and cancer [13, 62, 63]. However, a complete mechanistic understanding of SOX2 gene regulation by LncRNA SOX2‐OT has not been achieved. In this study, we demonstrate that SOX2‐OT genetic silencing causes decreased SOX2 expression in A549 lung cells. Additionally, an unexpected decrease of the expression of transcription factor GLI‐1, at both mRNA and protein levels, was also confirmed on A549 and NCI‐H1975 lung cells. Regarding SOX2‐OT functionality, previous studies have reported a ceRNA activity. Evidence suggests that SOX2‐OT is involved in promoting/protecting the positive expression/translation of SOX2 in bladder carcinomas, where SOX2‐OT has been mainly identified in the cytoplasm. Positive regulation of *SOX2* gene expression by LncRNA SOX2‐OT could also be the result of sponging activity targeting miR‐200c (miR200 subfamily members) [15], which has been detected in pancreatic ductal carcinomas [64]. On the other hand, during mouse cerebral cortex development, Sox2‐ot negatively regulates Sox2 expression, due to its interaction with the multifunctional transcription factor YY1, allowing its binding to promoter CpG islands within Sox2 gene locus [12]. Furthermore, our results suggest that LncRNA SOX2‐OT modulates the expression of the SOX2‐OT/SOX2/GLI‐1 trinomial axis, promoting changes in the histone code H3K4me3/H3K27Ac/H3K9me3/H3K27me3 at gene promoter sequences in lung cancer. However, additional studies will be necessary to confirm and validate epigenetic and nonepigenetic mechanisms involved in SOX2‐OT‐dependent SOX2‐OT/SOX2/GLI‐1 axis genetic expression.

Finally, we demonstrate that overexpression of the SOX2‐OT/SOX2/GLI‐1 trinomial axis, at transcripts/proteins levels in lung cancer patients, occurs independently of risk‐factor exposure (tobacco smoking/wood smoke exposure).

Similar results have reported a prognostic role for SOX2‐OT in malignant diseases, with a meta‐analysis which indicates that increased SOX2‐OT expression is significantly associated with poor overall survival (OS), and short disease‐free survival (DFS) in osteosarcoma and gastric cancer patients [32]. In the case of lung cancer, some studies have previously reported that SOX2‐OT overexpression is associated with worse clinical outcomes, suggesting a role as an independent prognostic factor, though the data remain inconclusive thus far [22, 32]. The MTA3/SOX2‐OT/SOX2 axis has been involved as a prognostic factor for poor clinical outcomes in esophageal squamous cell carcinoma patients [40]. Results from our study confirm that overexpressed LncRNA SOX2‐OT is involved in the control of SOX2‐OT/SOX2/GLI‐1 axis expression, as probable key archetypes, including as ‘scaffold’ to RNA‐binding protein (RBP) [65], and ceRNA function reported on SOX2‐targeting miR200c [15], or hypothetically involved in ceRNA activity on miR326 [66], or miR218 [67] whose miRNAs are involved in GLI‐1‐targeting. Some of these functional archetypes have been shown or suggested to participate in therapy resistance and clinical prognosis in lung cancer, as it has been previously reported [68]. In addition, recent evidences have demonstrated that, epigenetically, GLI1‐AS up‐regulates GLI‐1 gene expression [34], and is also associated with poor overall survival, and involved in failure response to cisplatin‐ and EGFR‐TKIs‐based therapy in lung cancer disease [28].

## 5 Conclusion

In lung cancer cells, the high co‐expression of the SOX2‐OT/SOX2/GLI‐1 axis occurs by epigenetic influence functionality in a histone code‐dependent manner at gene promoter sequences level. Likewise, LncRNA SOX2‐OT promotes a functional modulation of the EGFR‐AKT‐ERK expression‐activation, promoting resistance to cisplatin/EGFR‐TKI‐erlotinib therapy, determining poorer overall survival of lung cancer patients.

## Conflict of interest

The authors declare no conflict of interest.

## Author contributions

FA‐M designed the study. AMH‐S, IP‐A, LAL, NH‐C, RCC, PPV, ERV, and CGTI performed the experiments and analyzed data. FAM and IPA analyzed and interpreted data and wrote the manuscript. CMM, BOQ, JZ, and OA contributed to the systematical review of the published findings, collected, and provided clinical samples. All authors read and approved the final manuscript.

### Peer Review

The peer review history for this article is available at https://publons.com/publon/10.1002/1878‐0261.12875.

## Supporting information


**Fig. S1.** Changes in histone enrichment by oncological treatment (cisplatin/EGFR‐TKI‐erlotinib) at SOX2‐OT gene promoter sequences. Histone code changes on LncRNA SOX2‐OT gene promoter sequences (distal and proximal regions) by oncological treatment in lung cancer cells A549. Dark bar represents baseline condition, and white bar represents cisplatin‐TKI‐erlotinib treatment. Error bars represent mean ± standard deviation of three independent experiments.
**Fig. S2.** SOX2‐OT expression increased total and phosphorylated AKT protein levels. A) Relative expression of SOX2‐OT (variants 1 and 6), at baseline cellular conditions. B) Relative expression of the SOX2, and GLI‐1 mRNAs at cellular baseline conditions. C) Analysis of the SOX2, GLI‐1, AKT and ERK protein levels at baseline cellular conditions D) Total‐AKT and ERK protein levels under cisplatin/TKI‐erlotinib treatment. E) Changes in the phosphorylated AKT and ERK proteins levels under cisplatin (IC25: 2.76 µM) or TKI‐erlotinib (IC25: 10.10 µM) treatment. CisPlat: cisplatin, TKI‐ER: TKI‐erlotinib. Error bars represent mean ± SD of three independent experiments.
**Fig. S3.** Genetic silencing of LncRNA SOX2‐OT promotes increase of phosphorylated‐AKT/ERK protein levels but not in total‐AKT/ERK protein, as well as, decreases the relative expression of SOX2 and GLI‐1, in contrast to *EGFR* gene in lung cancer cells NCI‐H1975. A) Relative genetic expression of LncRNA SOX2‐OT variants 1 and 6, by genetic silencing assays siRNAs. B) Genetic silencing assays of the LncRNA SOX2‐OT promotes increase in both phosphorylated‐AKT and phosphorylated‐ERK protein levels in NCI‐H1975 lung cancer cells at baseline conditions, comparing with total‐AKT and ERK protein relative levels. Relative densitometric analysis identified changes in the SOX2 and GLI‐1 protein levels, using siRNAs anti‐LncRNA SOX2‐OT. Changes were GAPDH normalized. C) Genetic silencing of LncRNA SOX2‐OT promotes sensitivity to both treatments EGFR‐TKI and cisplatin in NCI‐H1975 lung cancer cells. Cellular response to TKI‐erlotinib, by silenced SOX2‐OT, using siSCRs. TKI‐erlotinib response by siSCR IC50 = 0.072 µM, siOT1 IC50 = 0.121 µM, siOT2 IC50 = 0.015 µM, and siMix IC50 = 0.018 µM. siSCR IC25 = 0.0003 µM, siOT1 IC25 = 0.0005 µM, siOT2 IC25 = 0.0001 µM, and siMix IC25 = 0.00004 µM. siSCR IC10 = 1.060e‐0.006 µM, siOT1 IC10 = 2.469e‐006 µM, siOT2 IC10 = 8.953e‐007 µM, and siMix IC10 = 1.201e‐007 µM. Cellular response to cisplatin, by silenced SOX2‐OT using siSCR. Inhibitory concentrations (ICs) were determinate as follows: siSCR group IC50 = 3.508 µM, siOT1 group IC50 = 0.026 µM, and siMix group IC50 = 0.690 µM. siSCR group IC25 = 0.132 µM, siOT1 group IC25 = 0.0003 µM, and siMix group IC25 = 0.0079 µM. siSCR group IC10 = 0.005 µM, siOT2 group IC10 = 3.939e‐006 µM, and siMix group IC10 = 8.991e‐005 µM. D) mRNA SOX2, GLI‐1 and EGFR genetic expression assays. Error bars represent the mean ± SD of three independent experiments. Unpaired *t‐*test (Two‐tailed) was used to compare to the control (siSCR) and combination groups *p < 0.05, ***p < 0.001, ****p < 0.0001.
**Fig. S4.** Map of the GLI‐1 gene promoter sequences. Genetic silencing assays of the LncRNA SOX2‐OT increases histone enrichment of the H3K4me3/H3K27Ac and reduce histone enrichment H3K9me3/H3K27me at SOX2‐OT gene promoter sequences on lung cancer cells. A) Changes in the enrichment of the histone H3K4me3 and H3K27Ac, and B) Changes in the enrichment of the histone repressive H3K9me3 and H3K27me3, on GLI‐1 gene promoter sequences.
**Fig. S5.** A) LncRNA SOX2‐OT expression level in lung carcinomas derived from (INER Cohort) patients, cut line indicates threshold of lower level expression: normal lung adjacent tissues (n = 5), and lung tumor tissues (n = 16). B‐C) Kaplan‐Meier survival analysis for lung carcinoma patients (INCan cohort) with low or high GLI1‐AS and GLI‐1 expression. Survival rate was calculated using the log‐rank test.
**Table S1.** Primer sequences for qPCR assays.
**Table S2.** Sequence for the SOX2‐OT siRNAs.
**Table S3.** Sequence for the ChIP‐qPCR Assays.
**Table S4.** Univariate analysis on clinical and oncological variables of lung cancer patients, associated with differential expression of the LncRNA SOX2‐OT.
**Table S5.** Univariate analysis on clinical and oncological variables of lung cancer patients, associated with differential expression of the SOX2 gene.
**Table S6.** Univariate analysis on clinical and oncological variables of lung cancer patients, associated with differential expression of the LncRNA GLI1‐AS.
**Table S7.** Univariate analysis on clinical and oncological variables of lung cancer patients, associated with differential expression of the GLI‐1 gene.

## Data Availability

The datasets in this study are available from the corresponding author upon reasonable request.
