# Supplementary material for: LncRNA SOX2‐OT regulates AKT/ERK and SOX2/GLI‐1 expression, hinders therapy, and worsens clinical prognosis in malignant lung diseases
Source: Mol Oncol. 2020 Dec 25;15(4):1110–29. doi: 10.1002/1878-0261.12875 (PMC8024737; doi:10.1002/1878-0261.12875)
Supplement: Supplementary file 1 — Fig. S1. Changes in histone enrichment by oncological treatment (cisplatin/EGFR‐TKI‐erlotinib) at SOX2‐OT gene promoter sequences. Histone code changes on LncRNA SOX2‐OT gene promoter sequences (distal and proximal regions) by oncological treatment in lung cancer cells A549. Dark bar represents baseline condition, and white bar represents cisplatin‐TKI‐erlotinib treatment. Error bars represent mean ± standard deviation of three independent experiments. Fig. S2. SOX2‐OT expression increased total and phosphorylated AKT protein levels. A) Relative expression of SOX2‐OT (variants 1 and 6), at baseline cellular conditions. B) Relative expression of the SOX2, and GLI‐1 mRNAs at cellular baseline conditions. C) Analysis of the SOX2, GLI‐1, AKT and ERK protein levels at baseline cellular conditions D) Total‐AKT and ERK protein levels under cisplatin/TKI‐erlotinib treatment. E) Changes in the phosphorylated AKT and ERK proteins levels under cisplatin (IC25: 2.76 µM) or TKI‐erlotinib (IC25: 10.10 µM) treatment. CisPlat: cisplatin, TKI‐ER: TKI‐erlotinib. Error bars represent mean ± SD of three independent experiments. Fig. S3. Genetic silencing of LncRNA SOX2‐OT promotes increase of phosphorylated‐AKT/ERK protein levels but not in total‐AKT/ERK protein, as well as, decreases the relative expression of SOX2 and GLI‐1, in contrast to EGFR gene in lung cancer cells NCI‐H1975. A) Relative genetic expression of LncRNA SOX2‐OT variants 1 and 6, by genetic silencing assays siRNAs. B) Genetic silencing assays of the LncRNA SOX2‐OT promotes increase in both phosphorylated‐AKT and phosphorylated‐ERK protein levels in NCI‐H1975 lung cancer cells at baseline conditions, comparing with total‐AKT and ERK protein relative levels. Relative densitometric analysis identified changes in the SOX2 and GLI‐1 protein levels, using siRNAs anti‐LncRNA SOX2‐OT. Changes were GAPDH normalized. C) Genetic silencing of LncRNA SOX2‐OT promotes sensitivity to both treatments EGFR‐TKI and cisplatin in NCI‐H1 [file MOL2-15-1110-s001.pdf]

Supplementary information

Table S1

**Table S1. Primer sequences for qPCR assays**		
**Gene (LncRNA)**	**Sequences**	**Position (pb)**
SOX2-OT (V1)	5’-GCTCGTGGCTTAGGAGATTG-3’<br>3’-CTGGCAAAGCATGAGGAACT-5’	Sense:926-945<br>Antisense:1022-1041
SOX2-OT (V6)	5´CCTCTTCTCAGGTGTAGATCACCTAT-3’<br>3’-AACAGAGCTGTCTTGTAAGTGAGG-5’	Sense: 285-311<br>Antisense: 376-398
GAS5	5´-AACTTGCCTGGACCAGCTTA-3’<br>3’-CAAGCCGACTCTCCATACCT-5’	Sense: 201-220<br>Antisense: 305-324
GLI1-AS	5´-AGGAGGAGCCCCTCACTAAA-3’<br>3’-CCTCCCGAAGTGATGAGATTA-5’	Sense: 542-561<br>antisense: 616-636
ZEB1-AS1	5´-TGCTTTGCTTGGTTAAGTGTG-3’<br>3’-ATTCGGTAGGCGACTGAAAA-5’	Sense: 1337-1357<br>Antisense: 1394-1413
UCA1	5´-CCCAAGGAACATCTCACCAA-3’<br>3’-GATGGTCCAAGGGGCTTC-5’	Sense: 362-381<br>Antisense: 440-457
**Gene (mRNA)**	**Sequences**	**Position (pb)**
GLI-1	5’-CCAGGAATTTGACTCCCAAG-3’<br>3’-GGCTTTGAAGGGCCTCAG-5’	Sense: 1173-1192<br>Antisense: 1279-1296
SOX2	5′-TGGGTTCGGTGGTCAAGT-3′<br>3’-TGATCATGTCCCGGAGGT-5′	Sense: 1155-1172<br>Antisense:1251-1268
ZEB1	5′-GCCAACAGACCAGACAGTGTT-3’<br>3’-TCTGCATCTGACTCGCATTC-5′	Sense: 523-543<br>Antisense:671-693
GAPDH	5’-CTCTGCTCCTCCTGTTCGAC-3’<br>3’-GCCCAATACGACCAAATCC-5’	Sense:4-23<br>Antisense: 106-122
U1	5’-CCCTGCTCCAGTCGCTATC-3’<br>3’-CCACGTCCGTCTGATTCC-5’	Sense: 235-253<br>Antisense: 308-325

Table S2

Table S2. Sequence for the SOX2-OT siRNAs		
**siRNA**	**siRNA sequence**	**Sequence alignment with SOX2-OT/mRNA**
siOT1	5'-CAAUCAACUCUGAGAUCCAtt-3'	*5´AGAGAAGCCAGGCAACA GCCATATTAAAGAAGAAAACAATCAACTCTGAGATCCAAC TTAGAAATAATGT-3´
siOT2	5'-CAAAAUAGGUCAUAGCAAAtt-3'	*5´GTCAAAATAGGTCATAGCAAAATGAACGTTTAAGATTAG GTTAAAACATTCCTCTGAAAT TTTAGTATTC-3´<br><br>**5´ATTACAAAACAGTCAAAATAGGTCATAGCAAAATGAACGT TTAAGATTAGGTTAAAACATTC CTCTGAAA-3´

*ID sequence: *NR_075091.1: SOX2-OT-V1 mRNA. **NR_075090.1: SOX2-OT-V6 mRNA

Table S3

Table S3. Sequence for the ChIP-qPCR Assays		
**Gene Promoter Region**	**Sequences**	**Genome Position (bp)**
GLI-1 (#6)	5´-TCGGACTCCTGACTTGAGGT-3´<br>5´-TCTTCTCCCCACCCAGTTCT-3´	-1830 -1673
GLI-1 (#5-4)	5´-CCAGCCTGGGCAAATAGTGA-3´<br>5´-TCAGAGACCCAGCTCAGTCA-3´	-1541 -1375
GLI-1 (#3)	5´-CCCTCCAGAACTTCGAGACG-3´<br>5´-GGCTCTGGAAGAAGGTGAGG-3´	-822 -665
GLI-1 (#2)	5´-TTCCATCCAAAGGGTGAGGC-3´<br>5´-CCCCGACAACCAGATTGAGG-3´	-612 -457
SOX2-OT Distal	5´-CTGCCTAATCATCCCACTCC-3´<br>5´-GAATGCCAGAGGAAAATCCA-3´	-2752 -2597
SOX2-OT Proximal	5´-GTGTGCCATGCCTACACAAT-3´<br>5´-GGCTTTTCAAAAAGGCTGAA-3´	-348 -193

Oligonucleotides (# numbers) have previously been reported MEOX2-binding domains additional references [1].

Additional References

1. Armas-López, L., Piña-Sánchez, P., Arrieta, O., Guzman de Alba, E., Ortiz-Quintero, B., Santillán-Doherty, P., Christiani, D. C., Zúñiga, J. & Ávila-Moreno, F. (2017) Epigenomic study identifies a novel mesenchyme homeobox2-GLI1 transcription axis involved in cancer drug resistance, overall survival and therapy prognosis in lung cancer patients, *Oncotarget.* 8.

Table S4. Univariate analysis on clinical and oncological variables of lung cancer patients, associated with differential expression of the LncRNA SOX2-OT.

**Variable**	**N=33**<br>**n (%)**	**SOX2-OT Dichotomous Expression Low *versus* High**	**p**
**Gender**<br>• Female<br>• Male	24 (72.7%)<br>9 (27.3%)	Low:11; High:13<br>Low: 5; High: 4	0.62
**Smoking history**<br>• No<br>• Yes	21 (63.6%)<br>12 (36.4%)	Low:9; High:12<br>Low:7; High:5	0.39
**Woodsmoke exposure**<br>• No<br>• Yes	22 (66.7%)<br>11 (33.3)	Low:11; High:11<br>Low:11; High:13	0.81
**Diabetes mellitus**<br>• No<br>• Yes	19 (57.6%)<br>14 (42.4%)	Low:8; High:11<br>Low:8; High:6	0.39
**Hypertension**<br>• No<br>• Yes	25 (75.8%)<br>8 (24.2%)	Low:11; High:14<br>Low:5; High:3	0.36
**ECOG**<br>• 0 or 1<br>• 2	26 (78.8%)<br>7 8(1.2%)	Low:13; High:13<br>Low:3; High:4	0.74
**Clinical stage**<br>• I – IIIA<br>• IIIB – IV	4 (12.2%)<br>29 (87.8%)	Low:2; High:2<br>Low:14; High:15	0.94
**CNS metastases**<br>• No<br>• Yes	17 (51.3%)<br>16 (48.5%)	Low:12; High:5<br>Low: 4; High:12	**0.009**
**Pleural effusion**<br>• No<br>• Yes	20 (60.6%)<br>13 (39.4%)	Low:8; High:12<br>Low:8; High:5	0.226
**Histology**<br>• Adenocarcinoma<br>• Other	29 (87.9)<br>4 (12.1)	Low:14; High:15<br>Low:2; High:2	0.95
EGFR<br>• WT<br>• del 19<br>• L858R<br>• Not reported	13 (39.4%)<br>11 (33.3%)<br>2 (6.1%)<br>7 (21.1%)	Low:5; High:8<br>Low:7; High:4<br>Low:0; High:2<br>Low:4; High:3	0.30
ALK<br>• WT<br>• Mutated<br>• Not reported	16 (48.5%)<br>5 (15.2%)<br>12 (36.4%)	Low:8; High:8<br>Low:1; High:4<br>Low:7; High:5	0.35
**Systemic 1st line therapy**<br>• Carbo/Cisplatin + Docetaxel<br>• EGFR-TKI<br>• Other (unknown)	17 (51.5%)<br>10 (30.3%)<br>6 (18.2%)	Low:9; High:8<br>Low:5; High:5<br>Low:2; High:4	0.70

Table S5. Univariate analysis on clinical and oncological variables of lung cancer patients, associated with differential expression of the SOX2 gene.

**Variable**	**N=33 n (%)**	**SOX2 Dichotomous Expression Low *versus* High**	***p***
**Gender**			
• Female	24 (72.7%)	Low:11; High:13	0.62
• Male	9 (27.3%)	Low:5; High: 4	
**Smoking history**			
• No	21 (63.6%)	Low:7; High:14	**0.021**
• Yes	12 (36.4%)	Low:9; High:3	
**Woodsmoke exposure**			
• No	22 (66.7%)	Low:11; High:11	0.81
• Yes	11 (33.3)	Low:5; High:6	
**Diabetes mellitus**			
• No	19 (57.6%)	Low:6; High:13	**0.024**
• Yes	14 (42.4%)	Low:10; High:4	
**Hypertension**			
• No	25 (75.8%)	Low:12; High:13	0.92
• Yes	8 (24.2%)	Low:4; High:4	
**ECOG**			
• 0 or 1	26 (78.8%)	Low:13; High:13	0.74
• 2	7 8(1.2%)	Low:3; High:4	
**Clinical stage**			
• I – IIIA	4 (12.2%)	Low:2; High:2	0.94
• IIIB - IV	29 (87.8%)	Low:14; High:15	
**CNS metastases**			
• No	17 (51.3%)	Low:11; High:6	0.055
• Yes	16 (48.5%)	Low: 5; High:11	
**Pleural effusion**			
• No	20 (60.6%)	Low:8; High:12	0.23
• Yes	13 (39.4%)	Low:8; High:5	
**Histology**			
• Adenocarcinoma	29 (87.9)	Low:13; High:16	0.26
• Other	4 (12.1)	Low:3; High:1	
EGFR			
• WT	13 (39.4%)	Low:7; High:6	
• del 19	11 (33.3%)	Low:5; High:6	0.52
• L858R	2 (6.1%)	Low:0; High:2	
• Not reported	7 (21.1%)	Low:4; High:3	
ALK			
• WT	16 (48.5%)	Low:8; High:8	
• Mutated	5 (15.2%)	Low:2; High:3	0.92
• Not reported	12 (36.4%)	Low:6; High:6	
**Systemic 1st line therapy**			
• Carbo/Cisplatin + Docetaxel	17 (51.5%)	Low:11; High:6	
• EGFR-TKI	10 (30.3%)	Low:3; High:7	0.16
• Other (unknown)	6 (18.2%)	Low:2; High:4	

Table S6. Univariate analysis on clinical and oncological variables of lung cancer patients, associated with differential expression of the LncRNA GLI1-AS.

**Variable**	**N=33**<br>**n (%)**	**GLI1-AS Dichotomous Expression Low *versus* High**	***p***
**Gender**<br>• Female<br>• Male	24 (72.7%)<br>9 (27.3%)	Low:10; High:14<br>Low: 6; High: 3	0.20
**Smoking history**<br>• No<br>• Yes	21 (63.6%)<br>12 (36.4%)	Low:9; High:12<br>Low:7; High:5	0.39
**Woodsmoke exposure**<br>• No<br>• Yes	22 (66.7%)<br>11 (33.3)	Low:13; High:9<br>Low:3; High:8	0.08
**Diabetes mellitus**<br>• No<br>• Yes	19 (57.6%)<br>14 (42.4%)	Low:8; High:11<br>Low:8; High:6	0.39
**Hypertension**<br>• No<br>• Yes	25 (75.8%)<br>8 (24.2%)	Low:11; High:14<br>Low:5; High:3	0.36
**ECOG**<br>• 0 or 1<br>• 2	26 (78.8%)<br>7 8(1.2%)	Low:10; High:16<br>Low:6; High:1	**0.026**
**Clinical stage**<br>• I – IIIA<br>• IIIB - IV	4 (12.2%)<br>29 (87.8%)	Low:2; High:2<br>Low:14; High:15	0.94
**CNS metastases**<br>• No<br>• Yes	17 (51.3%)<br>16 (48.5%)	Low:8; High:9<br>Low: 8; High:8	0.87
**Pleural effusion**<br>• No<br>• Yes	20 (60.6%)<br>13 (39.4%)	Low:9; High:11<br>Low:7; High:6	0.62
**Histology**<br>• Adenocarcinoma<br>• Other	29 (87.9)<br>4 (12.1)	Low:13; High:16<br>Low:3; High:1	0.26
EGFR<br>• WT<br>• del 19<br>• L858R<br>• Not reported	13 (39.4%)<br>11 (33.3%)<br>2 (6.1%)<br>7 (21.1%)	Low:5; High:8<br>Low:7; High:4<br>Low:0; High:2<br>Low:4; High:3	0.30
ALK<br>• WT<br>• Mutated<br>• Not reported	16 (48.5%)<br>5 (15.2%)<br>12 (36.4%)	Low:7; High:9<br>Low:0; High:5<br>Low:9; High:3	**0.016**
**Systemic 1st line therapy**<br>• Carbo/Cisplatin + Docetaxel<br>• EGFR-TKI<br>• Other (unknown)	17 (51.5%)<br>10 (30.3%)<br>6 (18.2%)	Low:9; High:8<br>Low:5; High:5<br>Low:2; High:4	0.70

Table S7. Univariate analysis on clinical and oncological variables of lung cancer patients, associated with differential expression of the GLI-1 gene.

Variable	N=33<br>n (%)	GLI-1<br>Dichotomous<br>Expression<br>Low *versus* High	*p*
**Gender**<br>• Female<br>• Male	 24 (72.7%)<br>9 (27.3%)	 Low:12; High:12<br>Low:4; High: 5	0.776
**Smoking history**<br>• No<br>• Yes	 21 (63.6%)<br>12 (36.4%)	 Low:10; High:11<br>Low:6; High:6	0.86
**Woodsmoke exposure**<br>• No<br>• Yes	 22 (66.7%)<br>11 (33.3)	 Low:11; High:11<br>Low:5; High:6	0.81
**Diabetes mellitus**<br>• No<br>• Yes	 19 (57.6%)<br>14 (42.4%)	 Low:10; High:9<br>Low:6; High:8	0.58
**Hypertension**<br>• No<br>• Yes	 25 (75.8%)<br>8 (24.2%)	 Low:14; High:11<br>Low:2; High:6	0.13
**ECOG**<br>• 0 or 1<br>• 2	 26 (78.8%)<br>7 8(1.2%)	 Low:11; High:15<br>Low:5; High:2	0.17
**Clinical stage**<br>• I – IIIA<br>• IIIB - IV	 4 (12.2%)<br>29 (87.8%)	 Low:0; High:4<br>Low:16; High:13	**0.038**
**CNS metastases**<br>• No<br>• Yes	 17 (51.3%)<br>16 (48.5%)	 Low:8; High:9<br>Low: 8; High:8	0.87
**Pleural effusion**<br>• No<br>• Yes	 20 (60.6%)<br>13 (39.4%)	 Low:8; High:12<br>Low:8; High:5	0.23
**Histology**<br>• Adenocarcinoma<br>• Other	 29 (87.9)<br>4 (12.1)	 Low:14; High:15<br>Low:2; High:2	0.95
EGFR<br>• WT<br>• del 19<br>• L858R<br>• Not reported	 13 (39.4%)<br>11 (33.3%)<br>2 (6.1%)<br>7 (21.1%)	 Low:6; High:7<br>Low:7; High:4<br>Low:0; High:2<br>Low:3; High:4	0.39
ALK<br>• WT<br>• Mutated<br>• Not reported	 16 (48.5%)<br>5 (15.2%)<br>12 (36.4%)	 Low:9; High:7<br>Low:1; High:4<br>Low:6; High:6	0.36
**Systemic 1st line therapy**<br>• Carbo/Cisplatin + Docetaxel<br>• EGFR-TKI<br>• Other (unknown)	 17 (51.5%)<br>10 (30.3%)<br>6 (18.2%)	 Low:9; High:8<br>Low:5; High:5<br>Low:2; High:4	0.71

Figure S1

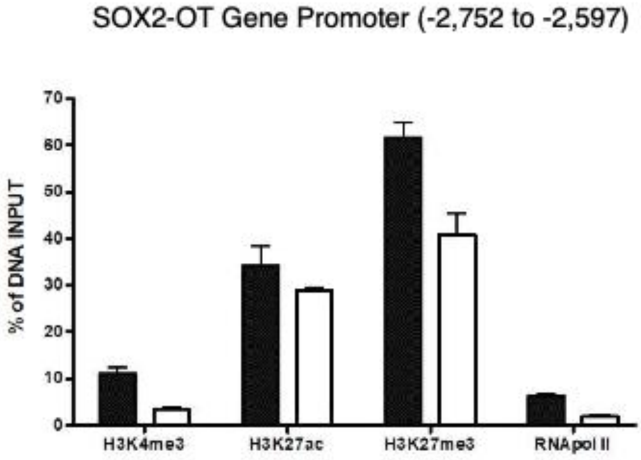

SOX2-OT Gene Promoter (-2,752 to -2,597)
% of DNA INPUT
70
60
50
40
30
20
10
0
H3K4me3
H3K27ac
H3K27me3
RNApol II

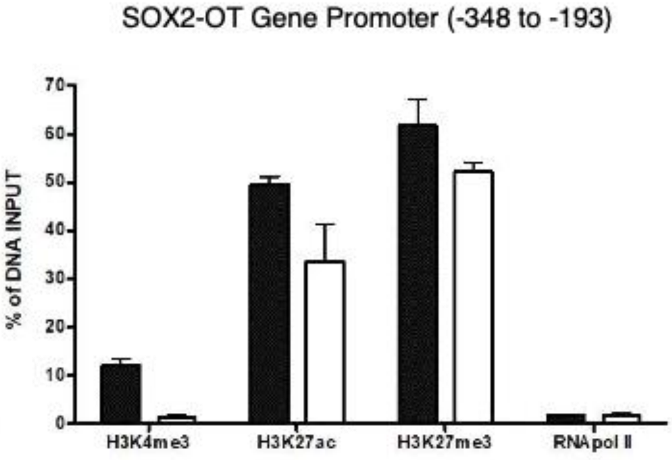

SOX2-OT Gene Promoter (-348 to -193)
% of DNA INPUT
70
60
50
40
30
20
10
0
H3K4me3
H3K27ac
H3K27me3
RNApol II

Figure S2

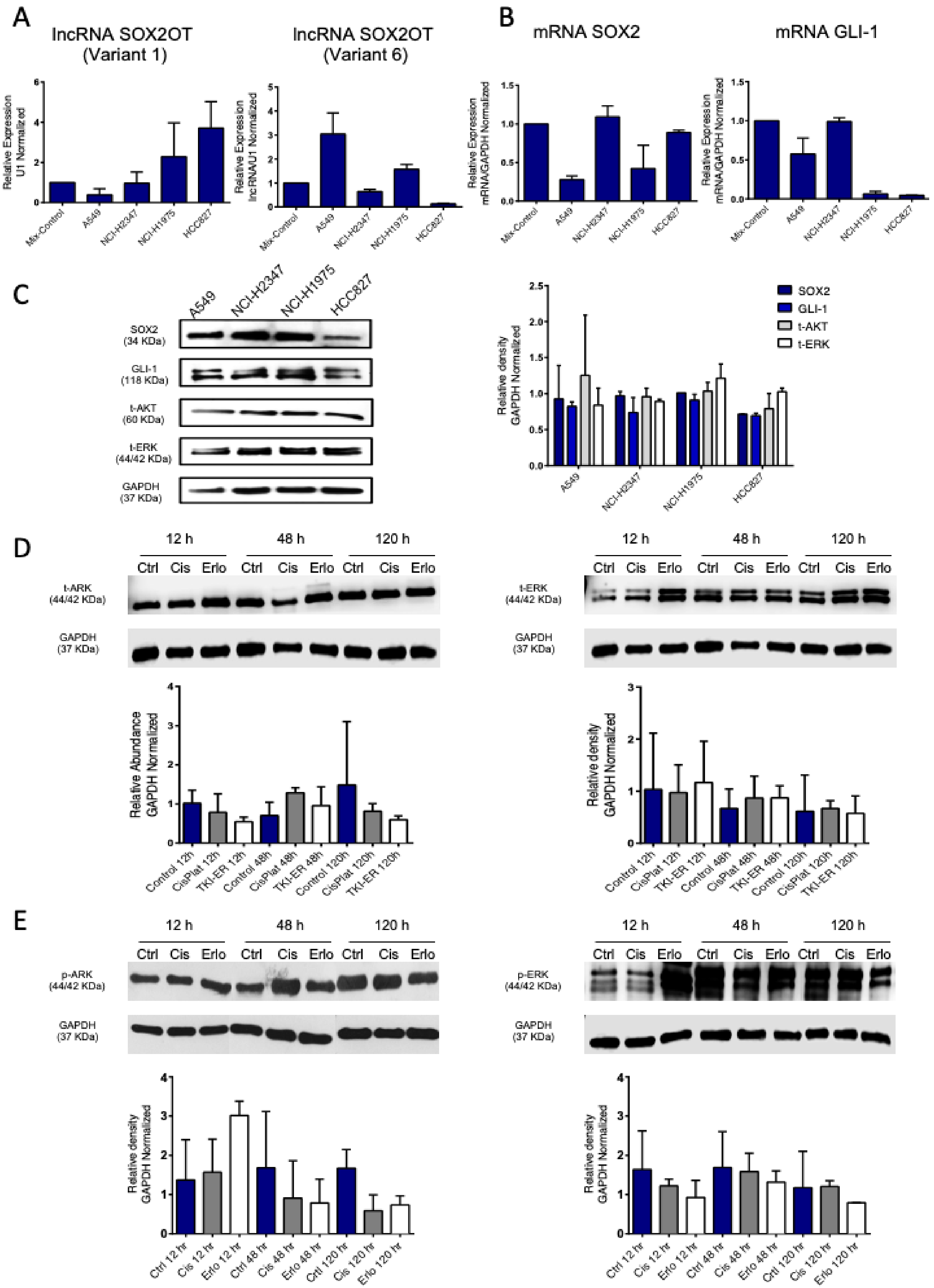

A
lncRNA SOX2OT (Variant 1)
lncRNA SOX2OT (Variant 6)
B
mRNA SOX2
mRNA GLI-1
Relative Expression U1 Normalized
Relative Expression lncRNA/U1 Normalized
Relative Expression mRNA/GAPDH Normalized
Mix-Control
A549
NCI-H2347
NCI-H1975
HCC827
C
SOX2 (34 KDa)
GLI-1 (118 KDa)
t-AKT (60 KDa)
t-ERK (44/42 KDa)
GAPDH (37 KDa)
SOX2
GLI-1
t-AKT
t-ERK
Relative density GAPDH Normalized
D
12 h
48 h
120 h
Ctrl
Cis
Erlo
t-ARK (44/42 KDa)
t-ERK (44/42 KDa)
GAPDH (37 KDa)
Relative Abundance GAPDH Normalized
Control 12h
CisPlat 12h
TKI-ER 12h
Control 48h
CisPlat 48h
TKI-ER 48h
Control 120h
CisPlat 120h
TKI-ER 120h
E
p-ARK (44/42 KDa)
p-ERK (44/42 KDa)
Ctrl 12 hr
Cis 12 hr
Erlo 12 hr
Ctrl 48 hr
Cis 48 hr
Erlo 48 hr
Ctrl 120 hr
Crtl 120 hr
Cis 120 hr
Erlo 120 hr

Figure S3

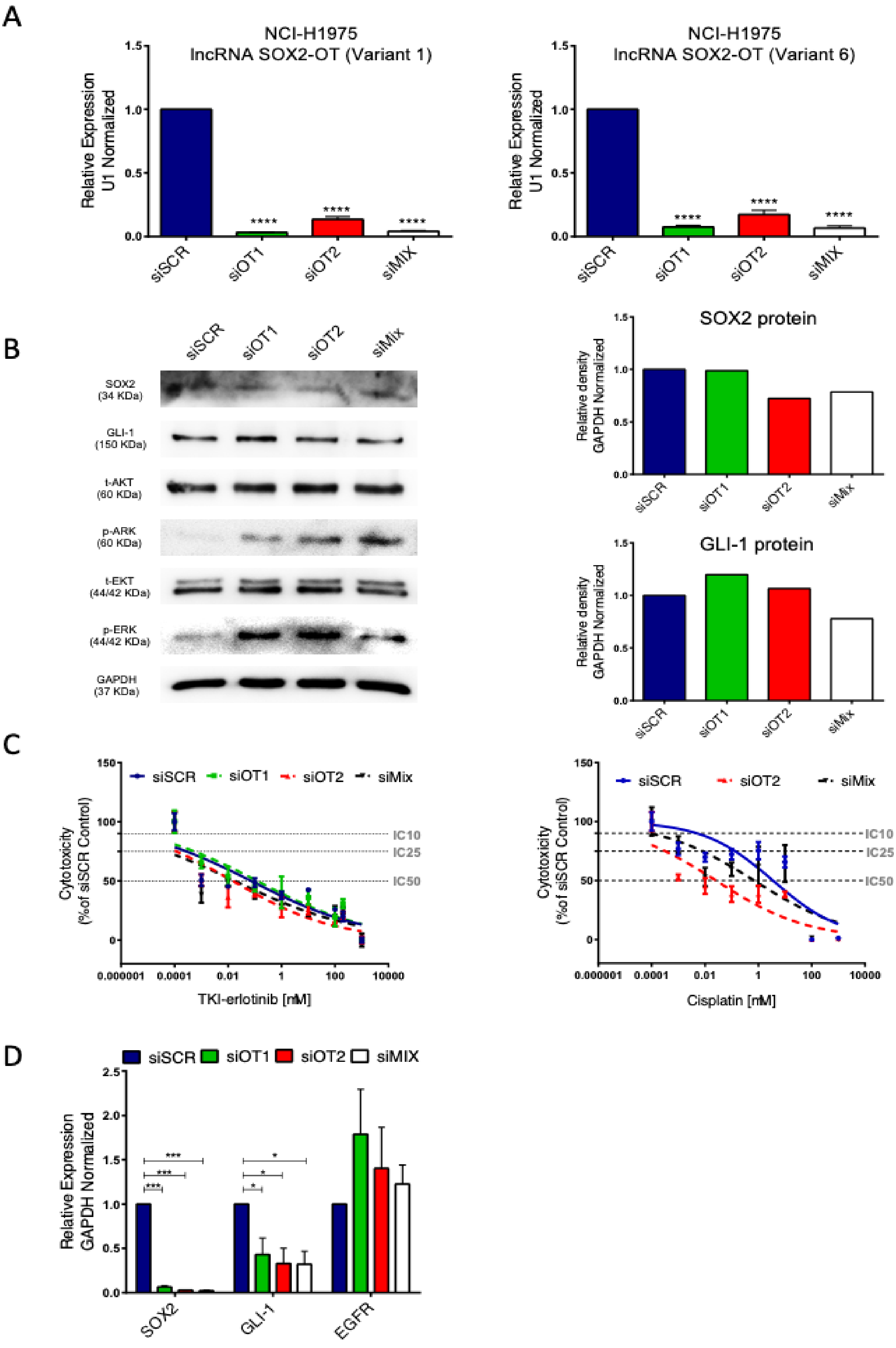
A
NCI-H1975
lncRNA SOX2-OT (Variant 1)
Relative Expression
U1 Normalized
****
siSCR
siOT1
siOT2
siMIX
NCI-H1975
lncRNA SOX2-OT (Variant 6)
B
siSCR
siOT1
siOT2
siMix
SOX2
(34 KDa)
GLI-1
(150 KDa)
t-AKT
(60 KDa)
p-ARK
(60 KDa)
t-EKT
(44/42 KDa)
p-ERK
(44/42 KDa)
GAPDH
(37 KDa)
SOX2 protein
Relative density
GAPDH Normalized
GLI-1 protein
C
Cytotoxicity
(%of siSCR Control)
IC10
IC25
IC50
TKI-erlotinib [nM]
Cisplatin [nM]
D
Relative Expression
GAPDH Normalized
SOX2
GLI-1
EGFR

Figure S4

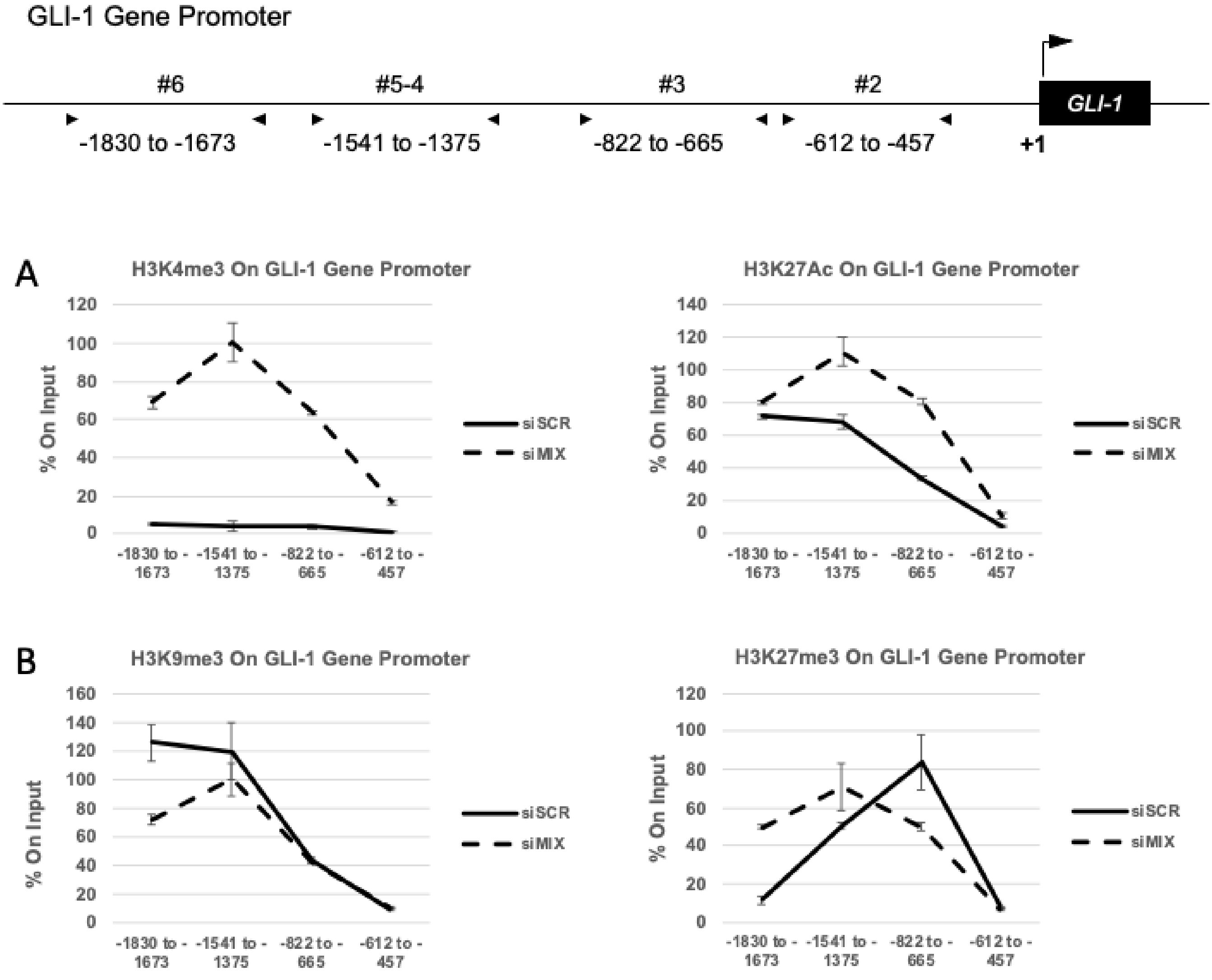
GLI-1 Gene Promoter
#6
#5-4
#3
#2
GLI-1
-1830 to -1673
-1541 to -1375
-822 to -665
-612 to -457
+1
A
H3K4me3 On GLI-1 Gene Promoter
% On Input
120
100
80
60
40
20
0
-1830 to -1673
-1541 to -1375
-822 to -665
-612 to -457
siSCR
siMIX
H3K27Ac On GLI-1 Gene Promoter
% On Input
140
120
100
80
60
40
20
0
-1830 to -1673
-1541 to -1375
-822 to -665
-612 to -457
siSCR
siMIX
B
H3K9me3 On GLI-1 Gene Promoter
% On Input
160
140
120
100
80
60
40
20
0
-1830 to -1673
-1541 to -1375
-822 to -665
-612 to -457
siSCR
siMIX
H3K27me3 On GLI-1 Gene Promoter
% On Input
120
100
80
60
40
20
0
-1830 to -1673
-1541 to -1375
-822 to -665
-612 to -457
siSCR
siMIX

Figure S5

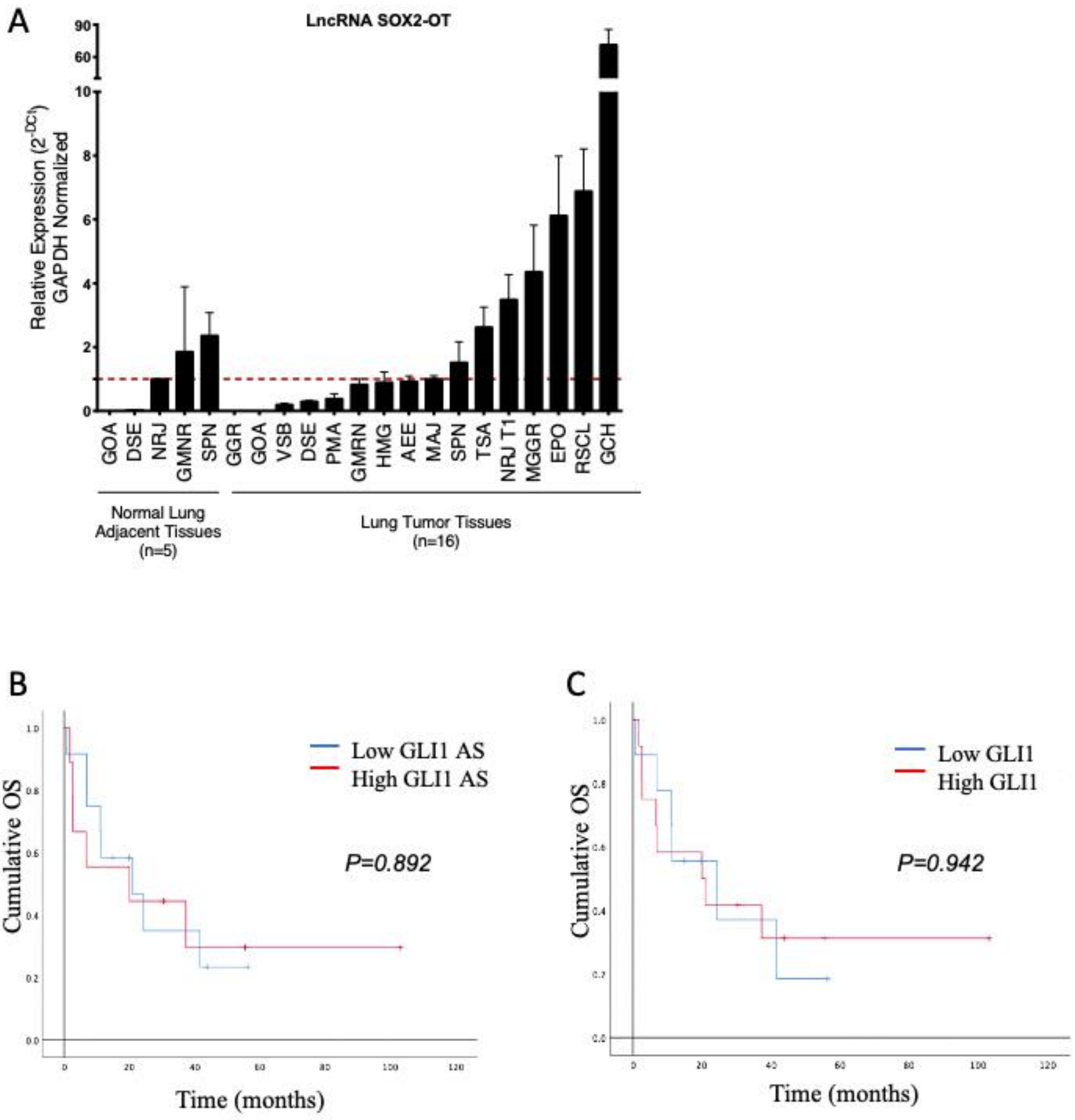
A
LncRNA SOX2-OT
Relative Expression (2-DCt)
GAPDH Normalized
GOA
DSE
NRJ
GMNR
SPN
GGR
GOA
VSB
DSE
PMA
GMRN
HMG
AEE
MAJ
SPN
TSA
NRJ T1
MGGR
EPO
RSCL
GCH
Normal Lung Adjacent Tissues (n=5)
Lung Tumor Tissues (n=16)
B
Cumulative OS
Low GLI1 AS
High GLI1 AS
P=0.892
Time (months)
C
Cumulative OS
Low GLI1
High GLI1
P=0.942
Time (months)
